# *Naja naja oxiana* Cobra Venom Cytotoxins CTI and CTII Disrupt Mitochondrial Membrane Integrity: Implications for Basic Three-Fingered Cytotoxins

**DOI:** 10.1371/journal.pone.0129248

**Published:** 2015-06-19

**Authors:** Sardar E. Gasanov, Indira H. Shrivastava, Firuz S. Israilov, Aleksandr A. Kim, Kamila A. Rylova, Boris Zhang, Ruben K. Dagda

**Affiliations:** 1 Applied Mathematics and Informatics Department, Moscow State University Branch, Tashkent, Uzbekistan; 2 Science Department, Tashkent Ulugbek International School, Tashkent, Uzbekistan; 3 Department of Computational and Systems Biology, University of Pittsburgh, Pittsburgh, Pennsylvania, United States of America; 4 Department of Pharmacology, University of Nevada School of Medicine, Reno, Nevada, United States of America; University of Pittsburgh School of Medicine, UNITED STATES

## Abstract

Cobra venom cytotoxins are basic three-fingered, amphipathic, non-enzymatic proteins that constitute a major fraction of cobra venom. While cytotoxins cause mitochondrial dysfunction in different cell types, the mechanisms by which cytotoxins bind to mitochondria remain unknown. We analyzed the abilities of CTI and CTII, S-type and P-type cytotoxins from *Naja naja oxiana* respectively, to associate with isolated mitochondrial fractions or with model membranes that simulate the mitochondrial lipid environment by using a myriad of biophysical techniques. Phosphorus-31 nuclear magnetic resonance (^31^P-NMR) spectroscopy data suggest that both cytotoxins bind to isolated mitochondrial fractions and promote the formation of aberrant non-bilayer structures. We then hypothesized that CTI and CTII bind to cardiolipin (CL) to disrupt mitochondrial membranes. Collectively, ^31^P-NMR, electron paramagnetic resonance (EPR), proton NMR (^1^H-NMR), deuterium NMR (^2^H-NMR) spectroscopy, differential scanning calorimetry, and erythrosine phosphorescence assays suggest that CTI and CTII bind to CL to generate non-bilayer structures and promote the permeabilization, dehydration and fusion of large unilamellar phosphatidylcholine (PC) liposomes enriched with CL. On the other hand, CTII but not CTI caused biophysical alterations of large unilamellar PC liposomes enriched with phosphatidylserine (PS). Mechanistically, single molecule docking simulations identified putative CL, PS and PC binding sites in CTI and CTII. While the predicted binding sites for PS and PC share a high number of interactive amino acid residues in CTI and CTII, the CL biding sites in CTII and CTI are more divergent as it contains additional interactive amino acid residues. Overall, our data suggest that cytotoxins physically associate with mitochondrial membranes by binding to CL to disrupt mitochondrial structural integrity.

## Introduction

Cobra venom cytotoxins, also known as cardiotoxins, are small basic amphipathic proteins (~60–62 amino acid residues) that belong to the large family of toxins containing three elongated finger-like loops including a divergent N-terminal loop (reviewed in [1]). Cytotoxins exhibit strong amphiphilicity on their molecular surface: apolar tips (loops I-III) that are flanked by small stretches of highly conserved positively charged Lys and Arg residues. In addition, cytotoxins are composed of two β-sheets that are stabilized by four conserved disulfide bonds [2]. Cytotoxins are predominantly characterized as cell membrane-permeabilizing proteins that can associate with anionic phospholipids located on the surface of the plasma membranes prior to their intracellular uptake. In addition to their ability to interact with cell membranes, recent evidence suggest that these proteins target the lipid bilayers of intracellular organelles including mitochondria (reviewed in [1]).

Cytotoxins have been classified as either S-type cytotoxins (contain a serine in amino acid position 28 of loop II) or as P-type cytotoxins (contain a proline in amino acid position 30 of loop II). Interestingly, this single amino acid substitution is correlated with drastic differences in cytotoxin-mediated cytotoxicity in different mammalian cell types. In most instances, P-type cytotoxins are generally more potent than S-type cytotoxins for disrupting cell membranes [3]. Pathological effects of cytotoxins in mammalian cells include their ability to depolarize excitable membranes of cardiac myocytes and neurons, deregulate the activity of cell membrane-bound enzymes and receptors, induce hemolysis and cytotoxicity, inhibit platelet aggregation, and bring about cardiac arrest (reviewed in [1]).

Cytotoxins electrostatically interact with anionic lipids on the cell membrane to form stable oligomeric complexes that can act as pore-forming structures [1]. Studies in phosphatidylcholine (PC) model membranes enriched with variable amounts of anionic phospholipids suggest that cytotoxins can disrupt the structure and molecular organization of phospholipid bilayers by dehydrating lipid bilayers [4], inducing the rapid aggregation of phospholipids [5], promote the formation of non-bilayer structures [1, 6], cause an intermembrane exchange of lipids [7] and cause the fusion of lipid bilayers [1, 5]. These molecular events are believed to underlie cytotoxin-mediated pathology including increased plasma membrane permeability and cell lysis.

Although cobra cytotoxins lack putative canonical mitochondrial and/or lysosomal targeting sequences, several cell biological studies suggest that certain cobra cytotoxins colocalize with mitochondria and lysosomes to cause mitochondrial swelling/fragmentation and depolarization and lysosomal permeabilization [1, 8–11]. For instance, treating cardiac myocytes with cytotoxins results in robust mitochondrial fragmentation and necrotic cell death while promoting mitochondrial permeability and induction of apoptosis of human leukemia K562 cells [12]. These observations suggest that unidentified mitochondrial biomolecules targeted by cytotoxins lead to either cytotoxin-mediated cell death through necrosis or bax-dependent apoptosis [9, 12].

Two particular cytotoxins known as CTI and CTII (S-type and P-type cytotoxins formerly known as Vc1 and Vc5 respectively [5, 13]) from *Naja naja oxiana* have been well characterized for their cytotoxic and cell membrane disrupting activities. While both cytotoxins show a high amino acid homology (80% homology), they exhibit major differences in cytotoxity towards different mammalian cell lines and in their abilities to disrupt liposomes containing different phospholipids [14]. These observations suggest that minor differences in their amino acid sequences or in their electrostatic environment between S- and P-type cytotoxins confer variable cytotoxicity.

Given that cytotoxins are highly basic amphipathic proteins [1], we surmised that cytotoxins selectively target mitochondria by interacting with anionic phospholipids such as cardiolipin (CL), a phospholipid enriched in inner mitochondrial membranes [15, 16]. In this study, we show for the first time that cobra venom cytotoxins CTI and CTII affect the structural organization of phospholipids in membranes of isolated mitochondria. To further understand the implications of cytotoxin-mediated structural alterations of mitochondrial membranes, we analyzed the abilities of CTI and CTII to interact with lipid films and large unilamellar liposomes enriched with cardiolipin (model membranes that simulate the outer mitochondrial membrane) by using a myriad of biophysical techniques including ^31^P-NMR spectroscopy, ^1^H-NMR spectroscopy, ^2^H-NMR spectroscopy, EPR of spin probes, luminescent probes, and differential scanning calorimetry. These biophysical studies were complemented with computer simulations of molecular docking analyses of several lipids including phosphatidylcholine, phosphatidylserine and cardiolipin (PC, PS and CL) with cytotoxins. Overall, this study suggests that CTI and CTII physically bind with cardiolipin (CL) in model membranes to alter the structural organization of lipid bilayers. Interestingly, CTII has a greater propensity for altering membranes enriched with phosphatidylserine (PS) and CL compared to CTI suggesting that minor differences in the amino acid sequence of the biological active loops dictate specificity for differentially binding to PS or CL. Docking simulations identified CL, PS and phosphatidylcholine (PC) binding sites in CTI and CTII. The putative docking sites for CL in CTI and CTII contain a cluster of N-terminal basic residues that are distinct from residues interacting with PS or PC. In summary, our data suggest that both cobra venom cytotoxins can associate with mitochondrial membranes to form non-bilayer structures prior to disrupting mitochondrial integrity.

## Materials and Methods

### Isolation of Mitochondria

Cauliflower mitochondria were isolated from 2.5 kg of the top layers (“rosettes”) as previously published [17]. Bovine heart mitochondria were isolated as previously described [18]. The final crude mitochondrial pellet was resuspended in 4.5 ml of washing medium (0.3 M mannitol, 10 mM MOPS, 1 mM EDTA, and 0.1% (w/v) BSA at pH 7.4) to a final concentration of 60 mg/ml and was used for the three independent ^31^P-NMR tests (1.5 ml of re-suspended mitochondria was placed in an NMR tube for one test). Following each mitochondrial isolation, the respiratory control index (RCI) values for all mitochondrial samples were measured as the ratio of the O_2_ consumption rate following the addition of ADP divided by the O_2_ consumption rate after all ADP has been converted to ATP [17]. Freshly isolated intact cauliflower and bovine heart mitochondria yielded similar RCI values (between 3 and 4) when succinate was employed as a substrate. The RCI values in all control samples were measured at 10°C every 30 min for 210 min, a time frame that is similar to the duration of ^31^P-NMR spectroscopy experiments. Cauliflower mitochondria showed no change in RCI values for 210 min while RCI values in bovine heart mitochondria declined after 90 min of incubation suggesting that bovine heart mitochondria undergoes structural/functional degradation by this time point. Hence, due to its more stable oxygen consumption activity, we decided to use cauliflower mitochondria for all ensuing ^31^P-NMR studies. Moreover, it is worth noting that we observed that the fluidity of cauliflower mitochondrial membranes at 10°C is equal to the fluidity of large unilamellar liposome membranes at 18°C [5, 6]. We thus conducted all experiments with large unilamellar liposomes at 18°C. All major biophysical-related equipment used in this study was equipped with sample temperature control devices.

To calculate the absolute amount of phospholipids contained in each mitochondrial sample, the integral intensity of the ^31^P-NMR signals from the experimental mitochondrial samples were normalized to the integral intensity of the ^31^P-NMR signals obtained from large unilamellar liposomes with known phospholipid concentrations used as standards. To study the effects of cytotoxins on mitochondria, mitochondrial samples for ^31^P-NMR studies were incubated with 9 × 10^−4^ M of either CTII or CTI. Each ^31^P-NMR experimental condition was repeated at least two times with technical replicates for each mitochondrial sample. The phospholipid concentration for each mitochondrial fraction analyzed was approximately 6.3 × 10^−2^ M with a variance of 8% for each ^31^P-NMR study.

### Molecular Reagents

Egg yolk L-α-phosphatidylcholine (PC), cardiolipin (CL) from *E*.*coli*, bovine brain L-α-phosphatidyl-L-serine (PS), dimiristoylphosphatidylcholine (DMPC), dipalmitoylphosphatidylcholine (DPPC), dimiristoylphosphatidylserine (DMPS), dipalmitoylphosphatidylserine (DPPS), the EPR spin labeled probes 5-doxylstearic acid (5-DSA), and potassium ferricyanide were all purchased from Sigma Chemical Co. (St. Louis, MO). All phospholipids were further purified on silica columns. *Naja naja oxiana* crude venom was obtained as a gift from Prof. L. Ya. Yukelson (Institute of Biochemistry, Uzbekistan Academy of Sciences). Cytotoxins CTII and CTI were isolated from 500 mg of crude venom according to a previously published procedure [19]. Cytotoxins were further purified by cation exchange HPLC by using a SCX 83-C-13-ET1 Hydropore column as previously described [20]. Pyrene, perylene and erythrosine (Sigma-Aldrich, UK) were used as phosphorescent probes while ferrocene (Sigma Chemical Co., St. Louis, MO) was used as a quencher of erythrosine phosphorescence.

### Preparation of unilamellar liposomes

Large unilamellar liposomes were prepared by using the ether evaporation method as previously described [21] in a buffer solution containing 10 mM Tris-HCl, pH 7.4, 0.5 mM EDTA, and 0.1 M NaCl but solubilized in water with different H isotopes (either ^1^H_2_O, ^2^H_2_O, or both) depending on the biophysical technique used. Liposomes characterized by ^1^H-NMR studies were prepared in a buffer containing ^2^H_2_O. Liposomes characterized by ^31^P-NMR spectroscopy were prepared in a buffer of 70% by volume of ^1^H_2_O and 30% ^2^H_2_O. The hydration of vacuum dried phospholipid films for ^2^H-NMR spectroscopy was done with ^2^H_2_O as previously published [4]. Liposomes containing natural phospholipids were maintained in an atmosphere predominantly composed of helium for 10 h at 10°C and for 5 h at 48°C for liposomes containing synthetic phospholipids. For most studies involving treatment with cytotoxins, PC liposomes contained 10 mol% of CL or PS, such concentration of anionic phospholipids is necessary to obtain the sufficient sensitivity to observe structural effects of cytotoxins on lipid bilayers as measured by most biophysical-related techniques employed for this study. Different liposomal samples (PC + 10 mol% CL or PS) were then treated with increasing concentrations of cytotoxins (CTI or CTII) prior to performing spectroscopy analyses as described below.

The minimum concentration of anionic phospholipids required to establish a biophysical interaction between PC liposomes and cytotoxins was analyzed by performing the phosphorescence assay of erythrosine under various concentrations of CL or PS and at a cytotoxin to lipid molar ratio of 0.01 as previously described [22]. For EPR studies, oriented multibilayer films were prepared by squeezing large unilamellar liposomes between two glass plates at a final phospholipid concentration of 50 mM as previously described [23]. The final concentrations of phospholipids used for ^1^H-NMR, ^2^H-NMR, ^31^P-NMR, differential scanning microcalorimetry and phosphorescence assays were set at 13.3, 15.0, 65.0, 2.6, and 3.1 mM respectively.

### EPR, ^2^H-NMR, ^1^H-NMR, ^31^P-NMR, differential scanning calorimetry, and phosphorescence assays

To study the effects of CTI and CTII on the molecular mobility, anisotropy, and orientation of the molecules in lipid bilayers, EPR spectra of the spin probe 5-DSA in membrane samples were recorded with a Varian E-4 spectrometer (USA) at modulation amplitudes not exceeding 2 × 10^−4^ T and with a resonator input power not exceeding 20 mW at 18°C. The analysis of the EPR spectra was done in terms of the *B/C* ratio [20] and the *S* parameter [24]. *B* is defined as the intensity of the low-field component while *C* is defined as the intensity of the central component of EPR spectra taken with respect to the magnetic field perpendicular to the bilayer normal. The formula used to calculate the *S* parameter was previously published [24]. Each sample treated with vehicle control or cytotoxins was prepared and analyzed by EPR in three technical replicates for each of three independent samples. The means and standard deviations of these measurements were plotted as experimental data points. The variation between the triplicates was observed to be less than 5%.

To determine whether CTI and CTII can permeabilize membranes containing anionic phospholipids, ^1^H-NMR spectra from large unilamellar liposomes containing the shift reagent, potassium ferricyanide K_3_[Fe(CN)_6_], and treated with vehicle control or with cytotoxins were recorded at 18°C using a Varian XL-200 spectrometer (USA) at an operating frequency of 200 MHz. The width of the 90° pulse was 8.7 μs, the relaxation delay was 50 μs and the acquisition time for free induction signal was 1 s. The measurements of the integral intensity of ^1^H-NMR signals from the N^+^(CH_3_)_3_ groups of PC were done in triplicate readings. The variation between the triplicates was observed to be less than 6%.

To study the effects of CTI and CTII on the hydration status of lipid bilayers, ^2^H-NMR spectra from membrane samples treated with vehicle control or with cytotoxins were recorded with a Varian XL-200 spectrometer (USA) at 18°C with the following parameters: an operating frequency at 30.7 MHz, the width of the 90° pulse at 15 μs, the sweep width at 5 kHz, and the distance between pulses at 0.2 s. The exponential function of the free induction decay with a broadening factor of 5 Hz was used.

To study the ability of CTI and CTII to directly associate and disrupt mitochondria, ^31^P-NMR spectra of isolated mitochondrial fractions were recorded at 10°C. The ^31^P-NMR spectra from large unilamellar liposomes were recorded at 18°C. The ^31^P-NMR spectra were recorded with a Varian XL-200 spectrometer (USA) at an operating frequency of 80.99 MHz. Proton decoupling was performed by continuous irradiation with the 90° pulse set at 12 μs and a power of 20 kHz, and the distance between pulses of 0.8 s. To improve the signal to noise ratio, the free induction decay was enhanced by applying an exponential function which yielded a 50-Hz line broadening of the spectra. To analyze for the existence of non-bilayer packed phospholipids, the saturation of the lamellar phase high-field resonance signal was achieved by applying a DANTE pulse train as previously described [25]. For ^31^P-NMR assays, mitochondrial samples and large unilamellar liposome samples for each experiment were prepared twice and the integral intensity measurements of ^31^P-NMR signals were performed three times for each sample. The technical variation between measurements was observed to be less than 8% for mitochondrial samples while the technical variation for large unilamellar liposome samples was observed to be less than 5%.

For studies that measured the effects of CTI and CTII on the intermembrane exchange and fusion of large unilamellar liposomes, calorimetric curves were monitored at a recording rate of 1°C per minute using a differential scanning microcalorimeter DASM-4 (Saint-Petersburg, Russia). The instrumental base line calibration mark was obtained by scanning at 50 mW, DT = 4, as previously described [26].

Large unilamellar liposomes for phosphorescence assays were incubated with 5 × 10^−6^ M erythrosine for 20 h followed by incubation with 10^−5^ M ferrocene for 2 h at 4°C. To remove unbound probes, the liposomes were chromatographed on a Sephadex G 50 column (1.8 × 50 cm) according to [22]. In liposomes used for phosphorescence assays, oxygen was enzymatically removed by glucose oxidase treatment. The analyzed liposome samples were contained in a square (10 mm) quartz chamber at 18°C. The erythrosine phosphorescence quenching by ferrocene in the presence or absence of cytotoxin (cytotoxin to lipid molar ratio of 0.01) was detected with a filter specific for a wavelength greater than 700 nm following excitation with a pulsed laser apparatus (Chernogolovka, Russia). The lifetime of the excited state of erythrosine was estimated as the time dependence of attenuation of the probe glow using semi-logarithmic coordinates. Samples for each data point were made and recorded in triplicate. The error of the erythrosine excited state lifetime was determined to be less than 5%.

### Molecular Docking

In order to analyze the interaction of cytotoxins with individual phospholipids and to identify potential phospholipid docking sites, the phospholipid head groups of CL, PS, and PC were docked with the solution NMR structures of CTI and CTII (PDB entries 1ZAD and 1CB9 respectively) by using the AutoDockVina Version 4.2 program [27]. The PDB coordinates of CL were extracted from the bovine heart oxidoreductase crystal structure bound to CL (PDB ID# 1V54). The PDB coordinates of phosphatidylcholine (PC) were extracted from the structure of PITP complexed to DOPC (PDB ID# 1T27) while the PDB coordinates of PS were extracted from the crystal structure of Tim-4 bound to PS (PDB ID# 3BIB). The lipids were further edited to remove the alkyl chains using Avogadro [28], and the overall charges were checked and energy minimized using AutoDock. The phospholipid “ligands”, which consisted only of the phospholipid head groups, contained rotatable bonds whereas the cytotoxins were kept as rigid molecules for each run. The overall molecular surface of CTI and CTII were considered for these docking studies (blind docking). A grid box was set up with following dimensions: center of x = 0.271; center of y = 0.855; center of z = 0.382; length of x = 100Å; length of y = 104Å; length of z = 80Å (104 Å for CTII). It is important to note that the grid box was large enough to do a “blind” dock by covering the entire surface of the cytotoxin and a phospholipid “ligand” for each molecular docking simulation. Following each Autodock run, the best nine docked conformations were analyzed for ionic, ion-polar, ion-hydrogen and hydrogen bond interactions between phospholipid polar head groups and charged and polar amino acids of cytotoxins by using Python Molecular Viewer (MGL Tools, The Scripps Research Institute).

*Statistics*- The means and standard errors (±SE) were calculated from at least three independent experiments. Two group comparisons were performed using Student’s *t*-test. Multiple group comparisons were done using one-way analysis of variance and Fisher’s LSD. Values of p<0.05 were considered significant.

## Results

**Fig 1:**
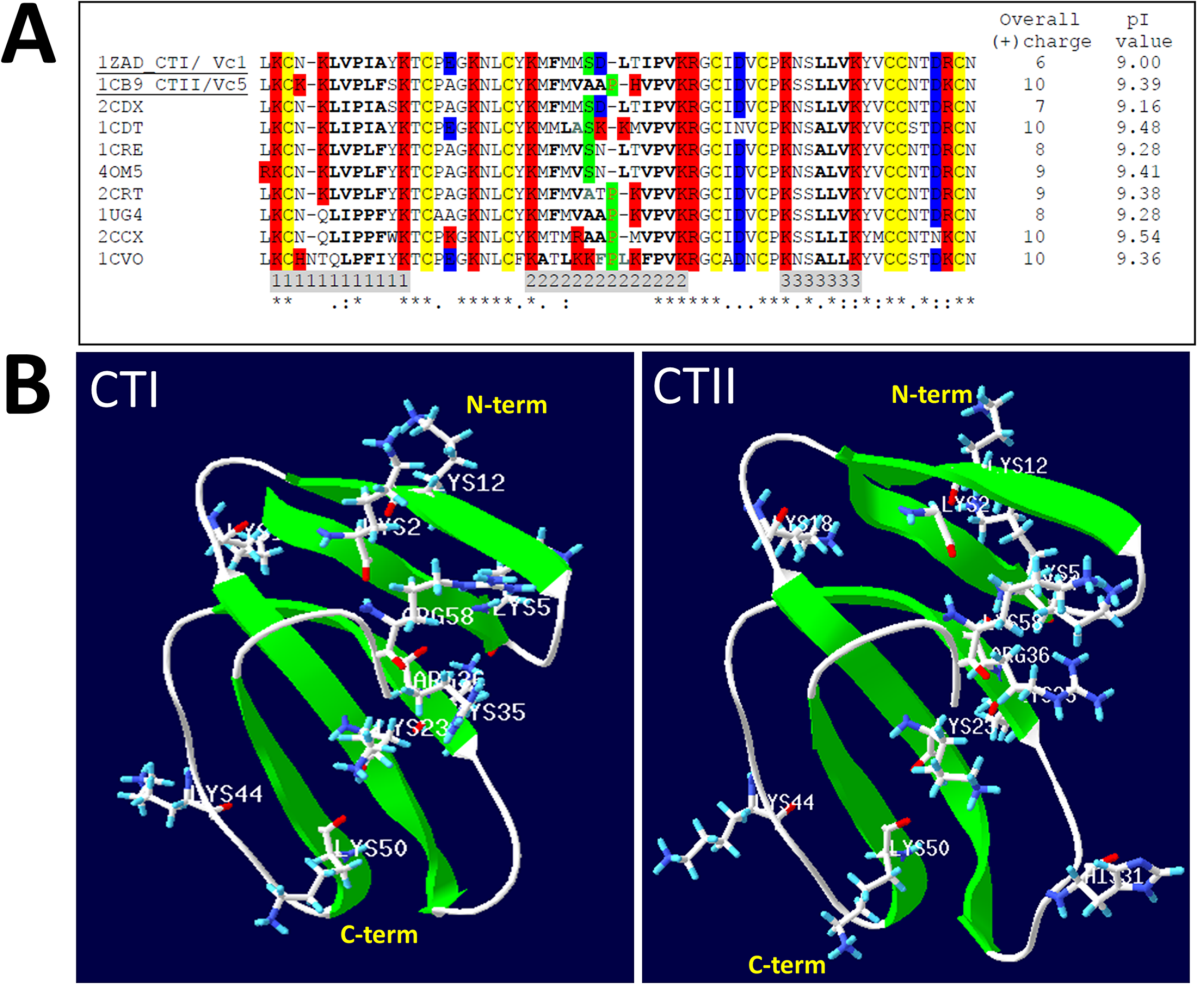
CLUSTAL 2.1 multiple amino acid sequence alignment of cobra venom cytotoxins. **A.** Amino acid sequence alignment of cobra venom cytotoxins, with resolved crystal structures, include 2CDX from *Naja naja atra*, 1ZAD (CTI/Vc1, underlined): *Naja naja oxiana*, 1CDT: *Naja mossambica mossambica*, 1CRE from *Naja naja atra*, 4OM5 from *Naja naja atra*, 2CRT from *Naja naja atra*, 1CB9 (CTII/Vc5, underlined) from *Naja naja oxiana*, 1UG4 from *Naja naja atra*, 2CCX from *Naja mossambica mossambica*, 1CVO from *Naja naja atra*. Conserved Cys residues are highlighted in yellow. Basic and acidic residues are highlighted in red and blue respectively. The amino acid residues located within each of the three loops (I-III) are highlighted in gray below the amino acid sequences. The variable residues Ser28 and Pro30 in loop 2, characteristic of S and P-type cytotoxins respectively, are highlighted in green. The hydrophobic residues of the three loops are bolded. The overall charge and isoelectric point (PI) value for each cytotoxin are shown in two columns to the right of the amino acid alignment. **B.** Ribbon diagrams of the crystal structures of CTI (PDB#1ZAD) and CTII (PDB#1CB9). All basic amino acid residues are represented as stick representations and labeled accordingly (Lys: lysine, Arg: arginine).

Cobra venom cytotoxins contain a highly conserved three-fingered fold. A high degree of hydrophobicity for loops I-III is associated with enhanced membrane disrupting activities of cytotoxins [29, 30] (Fig 1A and 1B).

A multiple amino acid sequence alignment of ten cobra venom cytotoxins suggests that the C-terminal region is highly conserved while a higher degree of variability is observed at the extremities and the tips of loops I-III (Fig 1A). Four cytotoxins possess an overall charge of +10. One of these four cytotoxins is an S-type (PDB ID #1CDT) cytotoxin while the remaining three cytotoxins are classified as P-type cytotoxins including CTII from *Naja naja oxiana* (PDB ID#1CB9), CTX IIb from *Naja mossambica mossambica* (PDB ID # 2CCX), and cytotoxin V from *Naja naja atra* (PDB ID# 1CVO). P-type cytotoxins show high degree of hydrophobicity for loop I compared to S-type cytotoxins (LIPPF or LVPLF for 2CCX and 1CB9 vs. LIPIA or LVPIA for 1CDT and 1ZAD). P-type cytotoxins also show stronger hydrophobicity for loops II (VAAP-VPV, AAP-VPV for 1CB9 and 2CCX) compared to S-type cytotoxins (ASK-VPV and MSD-IPV for 1CDT and 1ZAD) and in loop III as well (LLV and LLI for P-type cytotoxins vs. ALV for most S-type cytotoxins). Hence, the higher hydrophobicity and overall positive charge of CTII is consistent with its enhanced cytotoxic activities compared to CTI [13].

While recent cell biological studies have shown that cytotoxins can colocalize with mitochondria [31] and cause mitochondrial dysfunction [31], there is little evidence supporting a direct biophysical interaction of cytotoxins with mitochondrial membranes. In addition, the molecular mechanisms by which cytotoxins disrupt mitochondria are not known. CTI and CTII, are highly cationic three-fingered fold proteins. These proteins have a conserved three fingered domain that contains a cluster of basic amino acids located within the N-terminal region (Fig 1B). Hence, given their highly basic molecular surface, we surmised that CTI and CTII can directly associate with isolated mitochondrial fractions by binding to anionic phospholipids. Secondly, we hypothesize that the highly basic N-terminal region of CTI and CTII dock to CL to disrupt phospholipid bilayers.

### Cytotoxins interact with mitochondria to form non-bilayer structures

To test the hypothesis that CTI and CTII can bind to mitochondria, we employed ^31^P-NMR spectroscopy (a widely used biophysical technique to analyze protein-lipid interactions [32]) to study the interaction of cytotoxins with isolated mitochondrial fractions. For the purpose of this study, we employed cauliflower mitochondria instead of bovine heart mitochondria given their much longer structural stability following their isolation as assessed by oxygen consumption assays (described in Materials/Methods section). It is worth noting that plant and animal mitochondria are structurally similar [33] and have a similar content of mitochondrial phospholipids including CL, PS and PC [34, 35].

**Fig 2:**
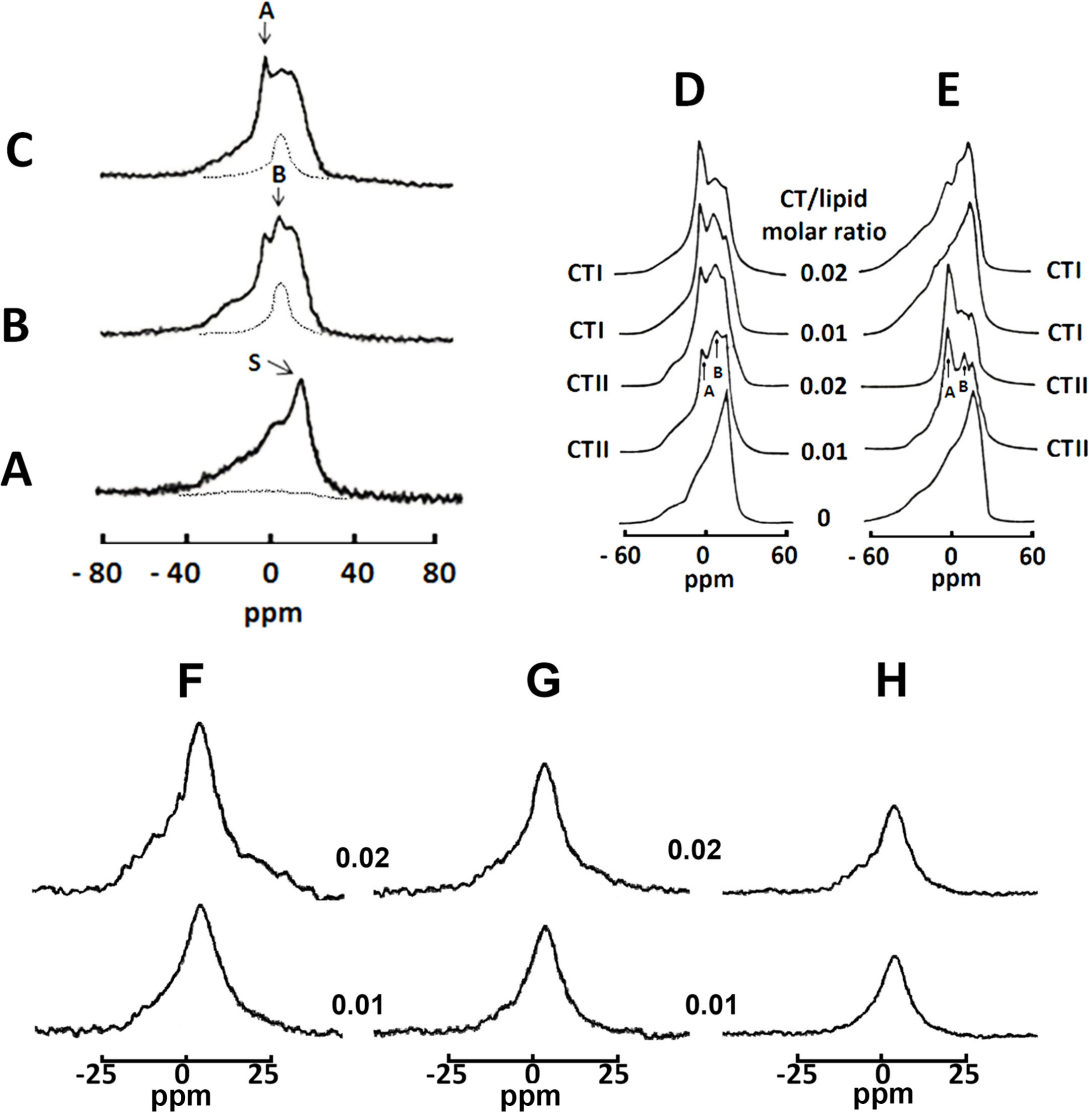
Effects of cobra venom cytotoxins CTII and CTI on isolated mitochondria and large unilamellar liposomes. ^31^P-NMR spectra of mitochondria or large unilamellar liposomes with different lipid compositions were monitored at 10°C. (**A**) ^31^P-NMR spectrum of a mitochondrial sample. (**B)** ^31^P-NMR spectrum of mitochondria treated with 9 × 10^−4^ M of CTII. (**C**) ^31^P-NMR spectrum of mitochondria treated with 9 × 10^−4^ M of CTI. Broken lines in A and B are saturation spectra observed after applying a DANTE train of saturation pulses at the high-field side of the lamellar spectrum (see arrow with letter **S**). ^31^P-NMR spectra of large unilamellar liposomes of PC+ 10 mol% CL (**D**) and of PC+ 10 mol% PS (**E**) monitored at 18°C and treated with the indicated cytotoxin-lipid ratios of CTII and CTI. All ^31^P-NMR data shown in this figure from isolated mitochondria and large unilamellar liposomes is representative of two independent experiments that showed similar results. Each sample was measured in triplicate readings. **F-H.** ^31^P-NMR spectra after applying a DANTE train of saturation pulses at the high-field peak of the lamellar spectrum of large unilamellar liposomes of PC+10 mol% CL treated with CTII (**F**) and CTI (**G**) and of PC+10 mol% PS treated with CTII (**H**) at the cytotoxin/lipid molar ratios of 0.01 (bottom spectra) and 0.02 (top spectra). Position of the signals in saturation spectra in **F**-**H** coincides with the position of ^31^P-NMR signal B.

In the absence of cytotoxins, proton-decoupled ^31^P-NMR from isolated mitochondria yielded a spectrum containing an asymmetrical shaped peak located at the high-field side (Fig 2A). This line-shape corresponds to an anisotropic orientation of phospho-ether bonds of phospholipids found in a lamellar phase, a property of biological lipid bilayers [32]. We then applied a DANTE train of saturation pulses at the high-field peak of the lamellar spectrum to determine whether signal(s) from non-lamellar phase(s) are hidden by the lamellar phase signal. Indeed, applying a DANTE saturation pulse led to the complete disappearance of the lamellar signals, indicating that all mitochondrial phospholipids in untreated mitochondrial samples are packed in a lipid bilayer (Fig 2A). On the other hand, incubating mitochondrial fractions with 9 × 10^−4^ M of CTI or CTII generated two additional ^31^P-NMR signals: signal A, a narrow peak located at 0 ppm and a broader signal B located to the right of signal A. Signal A is associated with a non-bilayer structure that exhibits rapid isotropic molecular mobility (1 x 10^−2^–10^−4^ s) [36]. Signal B, which is located between signal A and the highest peak of the lamellar signal, is also associated with non-bilayer structures but composed of phospholipids of restricted mobility [6]. We then investigated the molecular organization of the phospholipids associated with these two additional signals induced by treating mitochondria with CTI and CTII. Applying a DANTE train of saturation pulses led to a complete elimination of signal A and the signal associated with the lamellar phase of the^31^P-NMR spectra suggesting that phospholipids associated with the signal A efficiently exchange with phospholipids of a lamellar phase. On the other hand, signal B remained in the ^31^P-NMR spectra after applying the DANTE train of saturation (Fig 2B and 2C, hatched lines) indicating that these non-bilayer packed phospholipids do not exchange with a lamellar phase likely as a result of direct interaction with either CTII or CTI. We then quantitatively estimated the total amount of immobilized phospholipids induced by both cytotoxins by calculating the area under the curve below the ^31^P^-^NMR signal B after a DANTE train of saturation (Fig 2B and 2C, hatched lines) was applied. We calculated that at a lipid:toxin molar ratio 70:1, approximately 13.8 moles of phospholipids were immobilized by CTII while 10 moles of phospholipids were immobilized by CTI or 19.7% and 14.3% of the total mitochondrial phospholipids respectively. These results suggest that CTII can restrict the mobility of mitochondrial phospholipids to a greater extent than CTI. Overall, these data show that CTI and CTII can bind to mitochondrial membranes to form non-bilayer structures but differ in their ability to immobilize phospholipids.

### Cytotoxins interact with cardiolipin in large unilamellar liposomes

Mitochondrial membranes are predominantly composed of PC and a fraction of anionic phospholipids including CL and PS, which are predominantly found at the inner mitochondrial membrane [37]. On the other hand, CL exists as a minute fraction of the total phospholipid pool at the OMM. CL is predominantly restricted at membrane contact sites under physiological conditions and required for proper sorting/assembly of OMM-localized proteins [38]. To this end, we surmised that CTI and CTII are cationic proteins that can target CL in large unilamellar membranes (liposomes/lipid films). Unlike small sonicated liposomes, we used large unilamellar liposomes to simulate the lipid environment [24]. We analyzed the biophysical interaction of cytotoxins with large unilamellar liposomes composed of PC + CL (1:9 of CL: PC molar ratio) by using ^31^P-NMR. For comparative purposes, we also investigated the interaction of PC liposomes containing 10 mol% PS, a major anionic phospholipid of eukaryotic cell plasma membranes which is also found in small quantities in mitochondrial membranes [39].

The ^31^P-NMR spectra showed that large unilamellar liposomes composed of PC+10 mol% CL or of 10 mol% PS exclusively exist in a lamellar phase (Fig 2D and 2E, lower line shapes). However, treating PC+10 mol% CL liposomes with CTII and CTI resulted in the appearance of ^31^P-NMR signals A and B (Fig 2D, middle to upper line shapes) which correlate with non-bilayer structures [7] as seen in cytotoxin-treated mitochondrial fractions (Fig 2B and 2C). Interestingly, both ^31^P-NMR signals A and B were also observed in PC liposomes containing PS and treated with CTII (Fig 2E, fourth and fifth line shapes). On the other hand, the treatment of PC + PS liposomes with CTI did not produce ^31^P-NMR signals A and B (Fig 2E) suggesting that CTI and CTII differ in their ability to target PS but disrupt CL-containing membranes to the same extent. These results suggest that distinct molecular regions or lipid binding sites in the three-fingered domain of CTI and CTII are involved in binding CL and PS.

To obtain a better sense of affinity of CTI and CTII for phospholipids, we estimated the amount of phospholipids immobilized by cytotoxins in model membranes by calculating the area under the signal B of the ^31^P^-^NMR spectra remaining after applying a DANTE train of saturation at the high-field lamellar signal (Fig 2F–2H). Treating PC+ CL liposomes with CTII at toxin to lipid molar ratios of 0.01 and 0.02 led to an increase in the intensity and size of the ^31^P^-^NMR signals remaining after a DANTE saturation, whereas only a mild increase was observed in PC + PS liposomes (Fig 2F–2H). On the other hand, CTI produced a modest increase in immobilized phospholipids in PC + CL liposomes at the same toxin to lipid ratios (Fig 2G). These results suggest that CTII immobilizes a greater amount of phospholipids found in a non-bilayer phase compared to CTI. By calculating the integral of the intensity of these peaks, we estimated that approximately 16.5% and 24.4% of the total phospholipids were immobilized by CTII in PC+CL liposomes whereas 8.58% and 10.14% of the total phospholipids were immobilized by CTI at toxin to lipid molar ratios of 0.01 and 0.02 respectively. At the same time, an increase in the toxin to lipid molar ratio from 0.01 to 0.02 was associated with a decrease in the number of moles of immobilized phospholipids per mole of toxin (16.1 to 12.2 per mole of CTII and from 13.2 to 7.8 per mole of CTI). Similarly to PC+CL liposomes, in PC+PS liposomes an increase in CTII to lipid ratio from 0.01 to 0.02 was associated with a decrease of the number of immobilized lipids per mole of CTII from 9.4 to 5.1 (equal to 9.4% and 10.4% of total immobilized phospholipids for PC +PS liposomes). Therefore, these results suggest that CTII can immobilize a larger amount of phospholipids in large unilamellar liposomes that contain anionic phospholipids compared to CTI. The decrease in the number of immobilized lipids per mole of cytotoxin may be a direct consequence of a saturation of the lipid bilayer by CTI and CTII at high concentrations which leads to further mild increase in binding of the cytotoxin to phospholipid membranes or possible dimerization at high toxin to lipid molar ratios.

### Cytotoxins promote a disorientation of lipid bilayers containing cardiolipin

**Fig 3:**
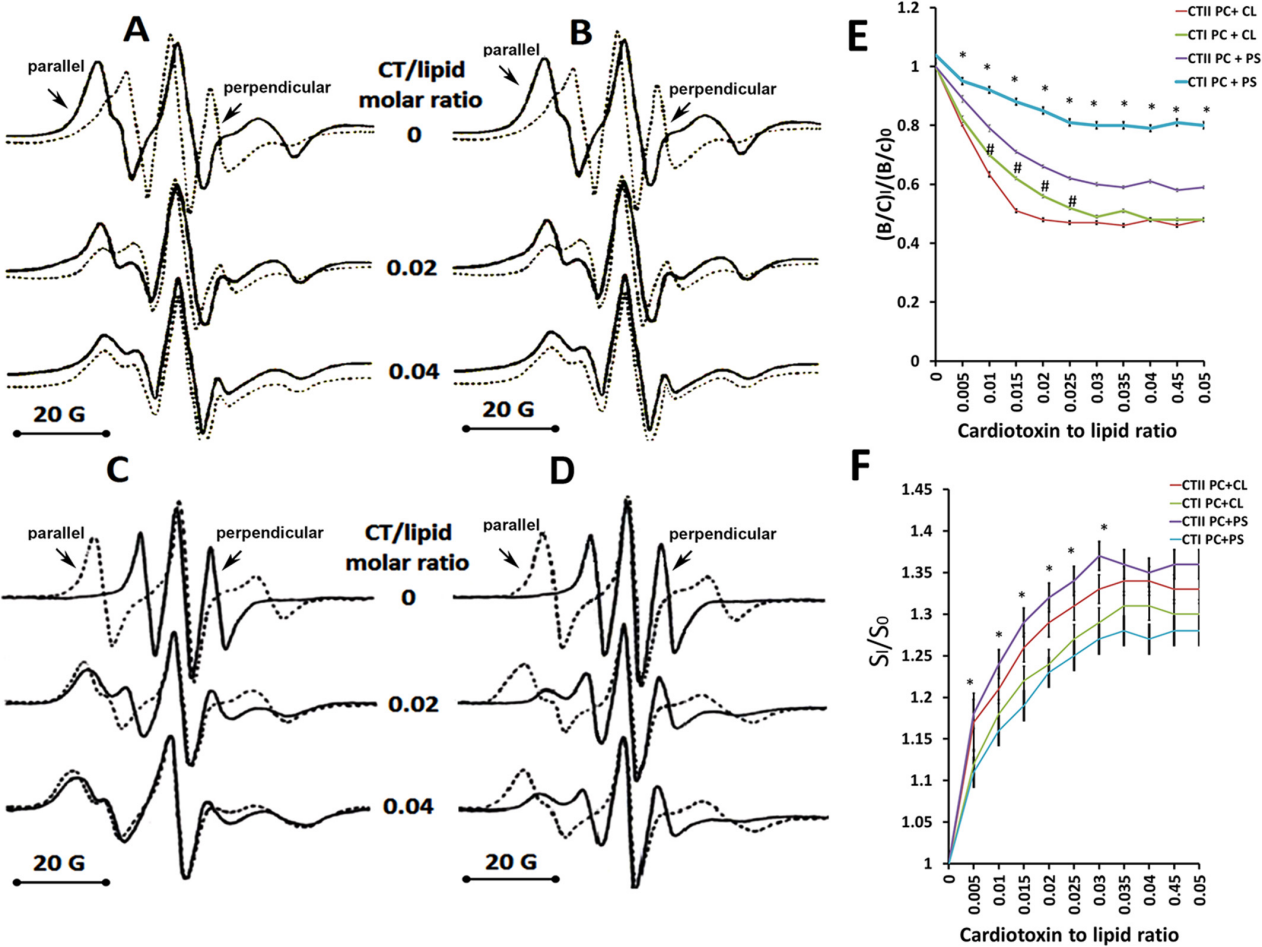
Effects of CTII and CTI on the orientation and organization of lipid bilayers. EPR spectra of 5-DSA in oriented multibilayer films of PC+10 mol% CL (**A, B**) or in multibilayer films of PC+10 mol% PS (**C, D**) containing 5-DSA: lipid molar ratio of 1:100 at 18°C. The magnetic field that is parallel or perpendicular to the bilayer normal is indicated by arrows pointed at the respective EPR spectral lines. Multibilayer films were treated with CTII (**A, C**) or with CTI (**B, D**) at the indicated cytotoxin to lipid molar ratios. The graph shown in **E** represents the means and standard errors of the B/C ratios of the EPR spectra compiled from three independent experiments involving PC+ CL or PC+ PS multibilayer films treated or untreated with the indicated cytotoxins (*:P<0.05 for PC + PS/CTII vs. PC + PS/CTI, #:p<0.05 for PC + CL/ CTII vs. PC +CL/CTI). The graph shown in **F** represents the means and standard errors of the S parameters of the EPR spectra obtained from three independent experiments involving PC+ CL or PC+ PS multibilayer films treated or untreated with the indicated cytotoxins (*:P<0.05 for PC + PS/CTII vs. PC + PS/CTI). Values for (*B/C*)_0_ and S_0_ represent mean B/C and S values from lipids without cytotoxins and values for (*B/C*)_I_ and S_I_ represent mean B/C and S values from lipids treated at various cytotoxin to lipid molar ratios.

To further characterize the biophysical effects of CTI and CTII on lipid bilayers, we employed EPR spectroscopy to analyze differences in the rotational movement and the orientation of phospholipids in cytotoxin-treated model membranes containing 5-doxylstearic acid (5-DSA). Unlike other biophysical techniques, EPR can quantify the spin and orientation of phospholipids at a higher temporal resolution (1 × 10^−6^–10^−11^ s) [40]. The orientation of lipid films analyzed at various angles of the magnetic field produces high resolution EPR spectra that facilitates the analysis of the packing order and mobility of lipids in model membranes [23]. Lipid bilayers are characterized by a strong anisotropic orientation of phospholipids. Indeed, the EPR spectra of 5-DSA in lipid films composed of PC+10 mol% CL showed strong spectral anisotropy as demonstrated by the presence of narrow EPR resonance lines obtained when the magnetic field is perpendicular to the bilayer normal and by the presence of wider resonance lines when the magnetic field is parallel to the lipid bilayer (Fig 3A and 3B upper spectra). PC+10 mol% CL lipid films treated with a low concentration of CTI and CTII produced a modest broadening and overlap of EPR spectra obtained when the magnetic field was applied perpendicular and parallel to the bilayer normal (Fig 3A and 3B; middle spectra). On the other hand, we observed a complete overlap of the EPR spectral lines when the magnetic field was parallel or perpendicular to the bilayer normal in PC+10 mol% CL films treated with the highest concentration of CTI or CTII (Fig 3A and 3B; lower spectra). The lack of anisotropy of these EPR spectra suggests a high degree of disorganization of phospholipids and a transition from a lipid bilayer structure to non-bilayer structures. Overall, these results suggest that both cobra venom cytotoxins are efficient at causing a disorganization of PC lipid films that contain CL.

On the other hand, we observed drastic differences on the effects of cytotoxins on the orientation and spin of phospholipids in PC+10 mol% PS lipid films treated with CTI or CTII. While CTII can cause a broadening and complete overlap of the EPR spectra analyzed at different orientations with respect to the magnetic field, treatment with CTI only produced a modest effect on the EPR line shape while retaining significant anisotropy (Fig 3C and 3D; middle and lower spectra). To quantitate the extent of the disorganization of lipid films induced by CTI and CTII, we calculated the *B/C* ratio and *S* parameter from the EPR spectra of cytotoxin-treated and untreated lipid films. The *B/C* ratio is very sensitive to macroscopic disordering [40] and to the formation of non-bilayer structures that exist in a lipid phase [23, 24]. The *S* parameter is sensitive to changes in the rotational movement of spin probes [24, 41]. In agreement with the EPR spectra, CTII and CTI induced a considerable decrease in *B/C* ratio values in PC+CL films whereas only CTII was able to significantly decrease the *B/C* ratio in PC+PS films compared to PC+PS films treated with CTI (Fig 3E). Both CTI and CTII significantly increased the *S* parameters in PC + PS and PC+ CL bilayers. However, a more robust effect of CTII on the *S* parameter was noted in PC + PS lipid films (Fig 3F). It should be noted that both the *B/C* ratio and *S* parameter values reach a plateau at cytotoxin to lipid ratio exceeding 0.03. It is conceivable that dimerization of cytotoxins may occur on the surface of liposomes at high concentrations of cytotoxins which can also lead to decreased phospholipid binding by cytotoxins as previously reported for a different cytotoxin[42]. Overall, the results obtained suggest that both cytotoxins can promote an aberrant transition of the lipid bilayer phase to a non-bilayer lipid phase in PC+CL films whereas only CTII significantly induced structural changes in PC+PS lipid films.

### Cytotoxins permeabilize and rupture membranes containing cardiolipin

**Fig 4:**
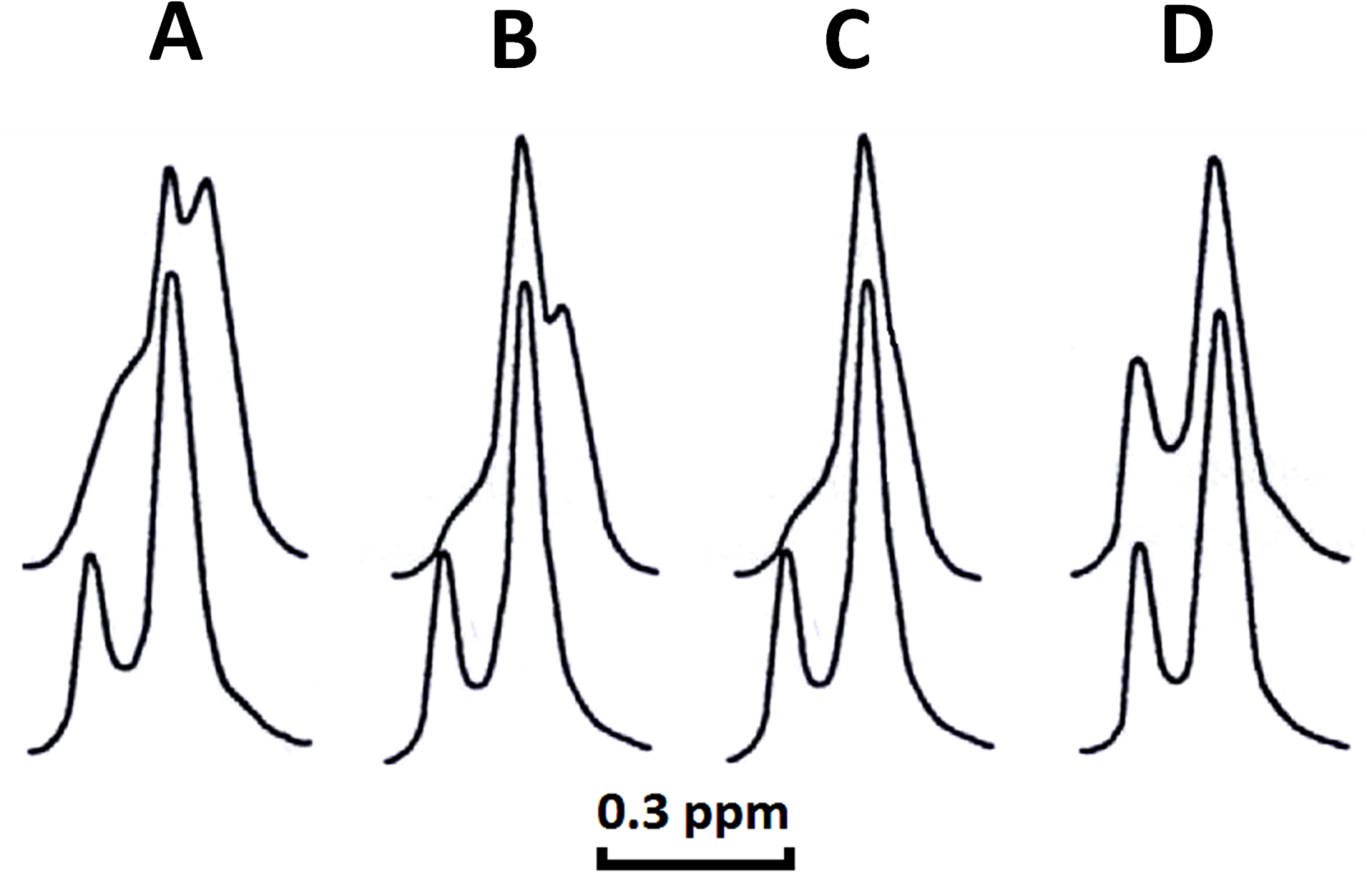
Cell permeabilizing activities of CTI and CTII on lipid bilayers. ^1^H-NMR spectra derived from the N^+^(CH_3_)_3_ groups of PC in large unilamellar liposomes composed of PC+ 10 mol% CL (**A** and **B**) or in PC+ 10 mol% PS (**C** and **D**) in the presence of potassium ferricyanide at 18°C and in the presence of CTII (**A** and **C,** top spectra) or of CTI (**B** and **D,** top spectra) at a cytotoxin/lipid molar ratio of 0.02. This figure shows a representative ^1^H-NMR traces from three independent experiments that showed similar results. Each sample (**A-D**) was measured in triplicate.

Next, we hypothesized that the formation of aberrant non-bilayer structures induced by CTI and CTII leads to the permeability and eventual rupture of lipid bilayers. To test this hypothesis, we performed ^1^H-NMR analysis of large unilamellar liposomes in a buffer containing potassium ferricyanide K_3_[Fe(CN)_6_] in the presence or absence of cytotoxins to analyze for the integrity of the inner and outer leaflets of the phospholipid membranes. Under basal conditions, the paramagnetic ion [Fe(CN)_6_]^-3^ does not permeate lipid bilayers of intact unilamellar liposomes [5]. The interaction of [Fe(CN)_6_]^-3^ with the N^+^(CH_3_)_3_ groups of PC molecules on the outer leaflet of liposomes shifts the ^1^H-NMR signal towards a higher magnetic field (right ^1^H-NMR signal). The smaller signal derived from the N^+^(CH_3_)_3_ groups of phospholipids of the inner leaflet is not shifted given that [Fe(CN)_6_]^-3^ cannot gain entry inside intact lipid bilayers. We found that treatment of PC+CL liposomes with both cytotoxins or of PC+PS liposomes with CTII caused the inner leaflet signal to disappear (Fig 4A–4C, upper spectra) and shift to the right of the NMR spectrum indicating that these lipid bilayers became permeable to [Fe(CN)_6_]^-3^ ions. On the other hand, PC+PS liposomes treated with CTI did not shift the position of the signal associated with the inner leaflet (Fig 4D, upper spectrum) indicating that the integrity of these liposomes was not compromised by CTI. A new ^1^H-NMR signal was observed on the high-field side of the ^1^H-NMR signal associated with the outer leaflet of the bilayer (Fig 4A and 4B, upper spectra) in PC+CL liposomes treated with both cytotoxins. However, the intensity of this additional signal in PC + CL liposomes treated with CTII (Fig 4A) was higher compared to liposomes treated with CTI (Fig 4B). The new high-field resonance in PC+CL liposomes treated by either CTII or CTI is likely a result of a closer distance between [Fe(CN_6_)]^-3^ ions and choline groups of PC which is associated with a non-bilayer structure that consists of PC molecules and CL molecules as previously described [4, 5]. Collectively, in agreement with other biophysical data, these results suggest that small structural differences in CTI and CTII confer differences in their ability to bind phospholipids (Fig 2D and 2E) and in disrupting membranes that model mitochondrial membranes while only CTII can disrupt lipid membranes containing PS.

### Cytotoxins dehydrate and fuse membranes containing cardiolipin

The observations that both CTI and CTII can induce the formation of non-bilayer structures and increase membrane permeability (Figs 2 and 4) suggest that these proteins promote the fusion of liposomes enriched with anionic phospholipids. To address this possibility, we employed ^2^H-NMR to determine whether CTII and CTI can induce the dehydration of PC+CL and PC+PS large unilamellar liposome surfaces given that membrane dehydration is an imperative phenomenon that precedes membrane fusion. The anisotropy of the mobility of ^2^H_2_O bound to membrane phospholipids results in a quadrupole splitting of a characteristic ^2^H-NMR resonance line. The extent of the splitting of this specific ^2^H-NMR resonance line is associated with increased anisotropy [43]. ^2^H-NMR spectrum of hydrated membranes composed of PC+10 mol% CL or PC+10 mol% PS (^2^H_2_O: lipid molar ratio = 10: 1) yields a signal with an axially symmetrical tensor of the electrical field gradient (S1 Fig). The quadrupole splitting values were observed at 1050 Hz and 1127 Hz for liposomes enriched with CL and PS respectively. However, these quadrupole splitting values were markedly smaller compared to immobilized water molecules in ice (~165kHz [44]). Treating PC+10 mol% CL hydrated membranes with CTI (^2^H_2_O: CTI: lipid = 100: 1: 10) yielded ^2^H-NMR spectra containing a superimposed duplet and singlet signals (S1 Fig). A singlet signal is associated with ^2^H_2_O molecules bound to cytotoxin suggesting that CTI immobilized ^2^H_2_O molecules. Moreover, the quadrupole splitting value of a duplet signal decreased from 1050 Hz to 700 Hz. A decrease in the quadrupole splitting value is associated with a large area of dehydrated membranes [44]. Hence, this significant decrease in quadrupole splitting value is a result of CTI-mediated dehydration of phospholipids that directly interact with the cytotoxin. Moreover, the value of quadrupole splitting in PC + 10 mol% CL membranes treated with CTII (^2^H_2_O: CTII: lipid = 100: 1: 10) was observed at 540 Hz (S1 Fig) suggesting that CTII has a greater effect on the fusion of PC+CL hydrated membranes compared to CTI. Similarly, CTI and CTII were able to dehydrate PC+PS membranes (S1 Fig). However, the extent of dehydration by CTII was significantly greater than CTI (quadrupole splitting values of 890 Hz and 590 Hz for CTI and CTII respectively).

**Fig 5:**
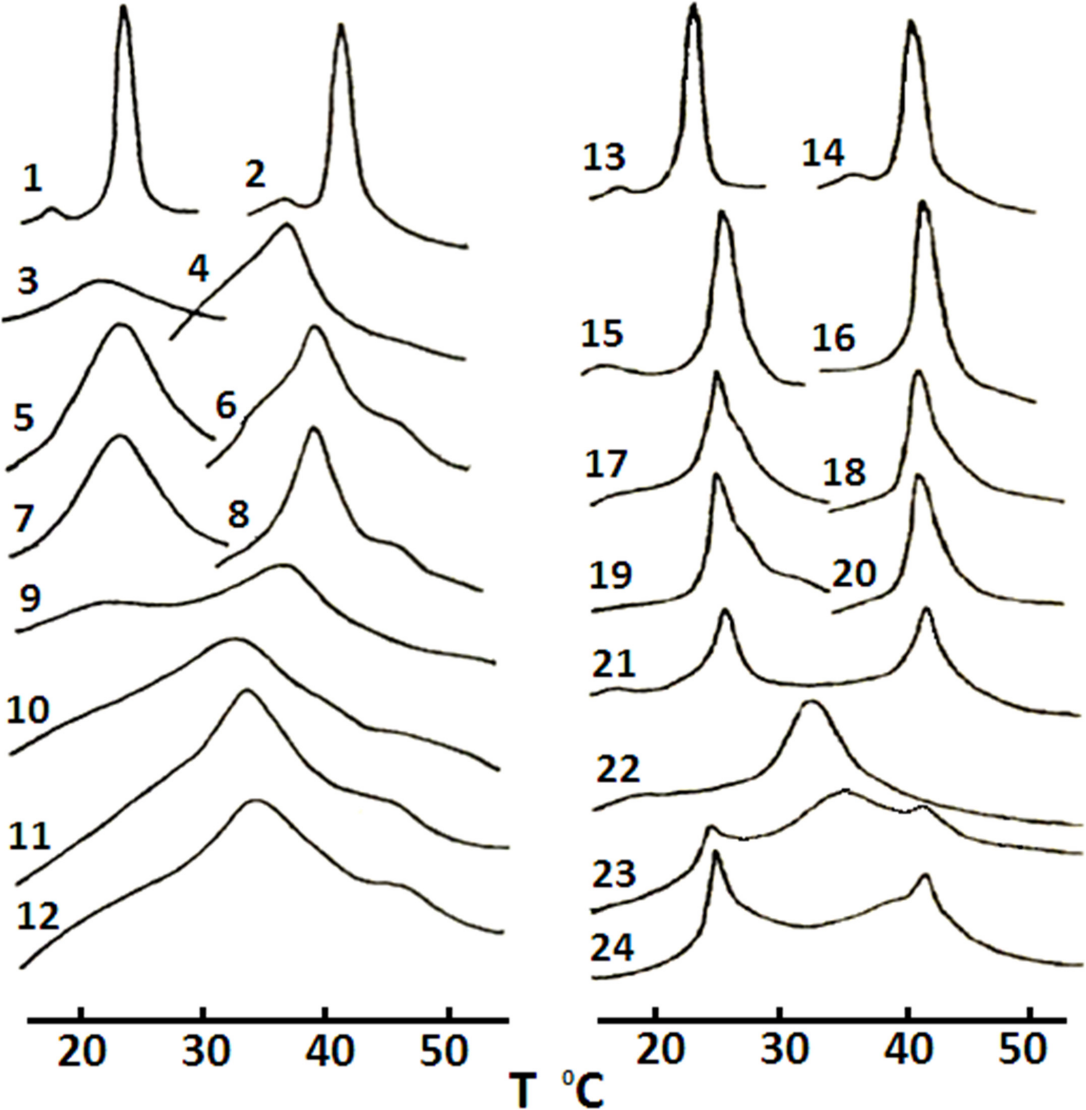
Fusogenic effects of CTI and CTII on lipid bilayers. Calorimetric curves of large unilamellar liposomes containing: DMPC alone (curves 1 and 13); DPPC alone (curves 2 and 14); DMPC+ 5 mol% CL (curve 3); DPPC+ 5 mol% CL (curve 4); DMPC + 5mol% CL + CTII (curve 5); DPPC + 5 mol% CL + CTII (curve 6); DMPC + 5 mol% CL + CTI (curve 7); DPPC +5mol% CL + CTI (curve 8); a mixture of DMPC + 5 mol% CL and DPPC + 5 mol% CL (curve 9); mixture 9 after sonication (curve 10); mixture 9 + CTII (curve 11); mixture 9 + CTI (curve 12); DMPC + 10 mol% DMPS (curve 15); DPPC + 10 mol% DPPS (curve 16); DMPC + 1 0mol% DMPS + CTII (curve 17); DPPC + 10 mol% DPPS + CTII (curve 18); DMPC + 10 mol% DMPS + CTI (curve 19); DPPC + 10 mol% DPPS + CTI (curve 20); a mixture of DMPC + 10 mol% DMPS and DPPC + 10 mol% DPPS (curve 21); mixture 21 after sonication (curve 22); mixture 21 + CTII (curve 23); mixture 21 + CTI (curve 24). The cytotoxin/lipid molar ratio was set at 0.02 for all experimental conditions. This figure shows representative calorimetric traces from three independent experiments that showed similar results. Each sample (curves 1–21) was measured in triplicate.

To further confirm the abilities of CTI and CTII to fuse lipid membranes, we analyzed the exchange of lipids between two populations of liposomes as a result of cytotoxin-induced intermembrane lipid exchange, a molecular event that is associated with membrane fusion [4, 45]. We used differential scanning microcalorimetry (DSM) to measure the heat flow produced by the intermembrane exchange of large unilamellar liposomes composed of different lipids. Large unilamellar liposomes made of a single type of saturated phospholipids undergo a solid to liquid phase transition within a narrow temperature range. The temperature value of a solid to liquid phase transition is correlated with the hydrocarbon chain length and the structure of the polar head of the phospholipids [5, 46]. In this study, we used two types of large unilamellar liposomes, one made of dimiristoylphosphatidylcholine (DMPC) and another of dipalmitoylphosphatidylcholine (DPPC). These two types of liposomes differ in values of their transition temperatures by approximately 18°C (Fig 5, curves 1–2 and 13–14). The addition of 5 mol% CL into liposomes composed of either DMPC or DPPC dramatically reduces the melting cooperativity as shown by a broadening of the calorimetric curves while shifting the calorimetric peaks toward lower temperatures (Fig 5, curves 3 and 4 respectively). The broadening of a calorimetric curve is particularly noticeable with DMPC, which possesses shorter alkyl chains compared to DPPC, thus making it more susceptible to lipid packing disordering (Fig 5, curve 3). The addition of CTII (Fig 5, curves 5 and 6) and CTI (Fig 5, curves 7 and 8) into DPPC+CL or DMPC+CL liposomes increased the lipid melting cooperativity in a similar trend as untreated DMPC and DPPC liposomes lacking CL (Fig 5, curves 1 and 2). This phenomenon is a result of cytotoxin-mediated phase segregation of CLs that frees areas of pure DMPC and DPPC. A calorimetric curve from a mixture of two populations of untreated liposomes (Fig 5, curve 9) demonstrated no evidence of inter-liposomal exchange since their individual maximal transition temperatures coincided with the maximal transition temperatures of curves 3 and 4 (Fig 5). As a positive control for this assay, the sonication of two liposome populations resulted in a single phase transition peak (Fig 5, curve 10). These results suggest that two populations of liposomes are completely mixed in a single set of liposomes. A similar result was obtained when CTII (Fig 5, curve 11) or CTI (Fig 5, curve 12) were added to a mixture of two liposomal populations. However, for these treatments, we observed more pronounced phase transition peaks for liposomal samples treated with CTII (Fig 5, curve 11) or CTI (Fig 5, curve 12) compared to sonication-mediated fusion of liposomes (Fig 5, curve 10), which was likely a result of cytotoxin-induced segregation of CL. Moreover, single transition peaks (Fig 5, curves 11 and 12) indicate that most of DMPC and DPPC are mixed, which most likely is a result of cytotoxin-induced fusion of liposomes [4, 6, 7, 45]. Similarly, both cytotoxins were found to slightly lower the transition phase temperatures of the calorimetric curves of DPPC or DMPC liposomes containing one of two types of PS molecules, dimiristoylphosphatidylserine (DMPS) and dipalmitoylphosphatidylserine (DPPS) (Fig 5, curves 17–20) compared to untreated liposomes (Fig 5, curves 15 and 16). The sonication of these two mixtures of liposomes results in the formation of a single phase transition peak (Fig 5, curve 22). The addition of only CTII, but not CTI, significantly broadens the calorimetric curves of a mixture of DPPC and DMPC liposomes containing DPPS and DMPS respectively (Fig 5, 23 and 24 respectively) compared to an untreated mixture of liposomes (Fig 5, curve 21). Overall, these results suggest that CTI and CTII can equally cause the inter-membrane exchange of lipids and fusion of model membranes containing CL, while only CTII can efficiently cause the inter-membrane exchange of lipids of PS containing liposomes as induced by cytotoxins. Collectively, so far our data suggest that minor structural elements found in P-type cytotoxins (CTII), but not in S-type cytotoxins (CTI), confer enhanced ability to remodel and disrupt membranes by permeabilizing bilayers (PS or CL), forming non-bilayer structures and dehydrating the surface of membranes, events associated with fusion of membranes.

### Minimal concentration of CL and PS needed for interacting with CTI and CTII

So far, large unilamellar PC liposomes employed in this study contained 10 mol% of CL or PS. This concentration of CL or PS exceeds the amount of anionic phospholipids found in the OMM under physiological conditions. Hence, in order to elucidate the minimal concentrations of CL and PS needed to interact with CTI and CTII in PC liposomes, the quenching of erythrosine phosphorescence induced by ferrocene was analyzed for various liposomal membrane preparations at a cytotoxin to lipid molar ratio of 0.01. The phosphorescence life-time value depends on the rate of molecular movement of a probe in the lipid phase and is very sensitive to the interaction of cytotoxins with the surface of liposomes [22]. By utilizing this assay, we showed that cytotoxins can bind to PC liposomal membranes containing CL at a minimum concentration of 2 mol% for CTII and 3 mol% for CTI (S2 Fig). The minimum concentration of PS required for binding to CTII in PC + PS liposomes is 8 mol% PS and 9 mol% PS for CTI (S2 Fig). These findings suggest that both CTII and CTI bind to CL at an approximate concentration as found in OMM [37, 39]. Moreover, these results suggest that both cytotoxins have a higher affinity for anionic phospholipids compared to PC (S2 Fig, 1% CL or PS).

### Molecular docking simulations identified interacting residues in CTI and CTII that bind to CL

So far, our data suggest that minor differences in the primary or molecular surface of CTII confer increased abilities to disrupt membranes containing CL or PS compared to CTI. To address this hypothesis, we performed molecular docking simulations to identify molecular mechanisms by which CTI and CTII bind to CL, PC or PS. Hence, to identify putative binding sites of phospholipids on the molecular surfaces of CTI and CTII, we separately docked the PC, PS and CL to the solution NMR structures of CTI and CTII. Given that alkyl chains of phospholipids are confined inside the lipid bilayer and do not likely interact with the cytotoxin at the initial stage of cytotoxin binding to membrane surface, we disregarded the alkyl chains of phospholipids for the docking studies by truncating the alkyl chains. Hence, the alkyl chains were truncated, energy minimized in Autodock and verified for the correct overall charge for PC, PS and CL. It is expected that this type of docking technique yields potential docking sites with more accuracy and higher resolution as previously described [47].

**Fig 6:**
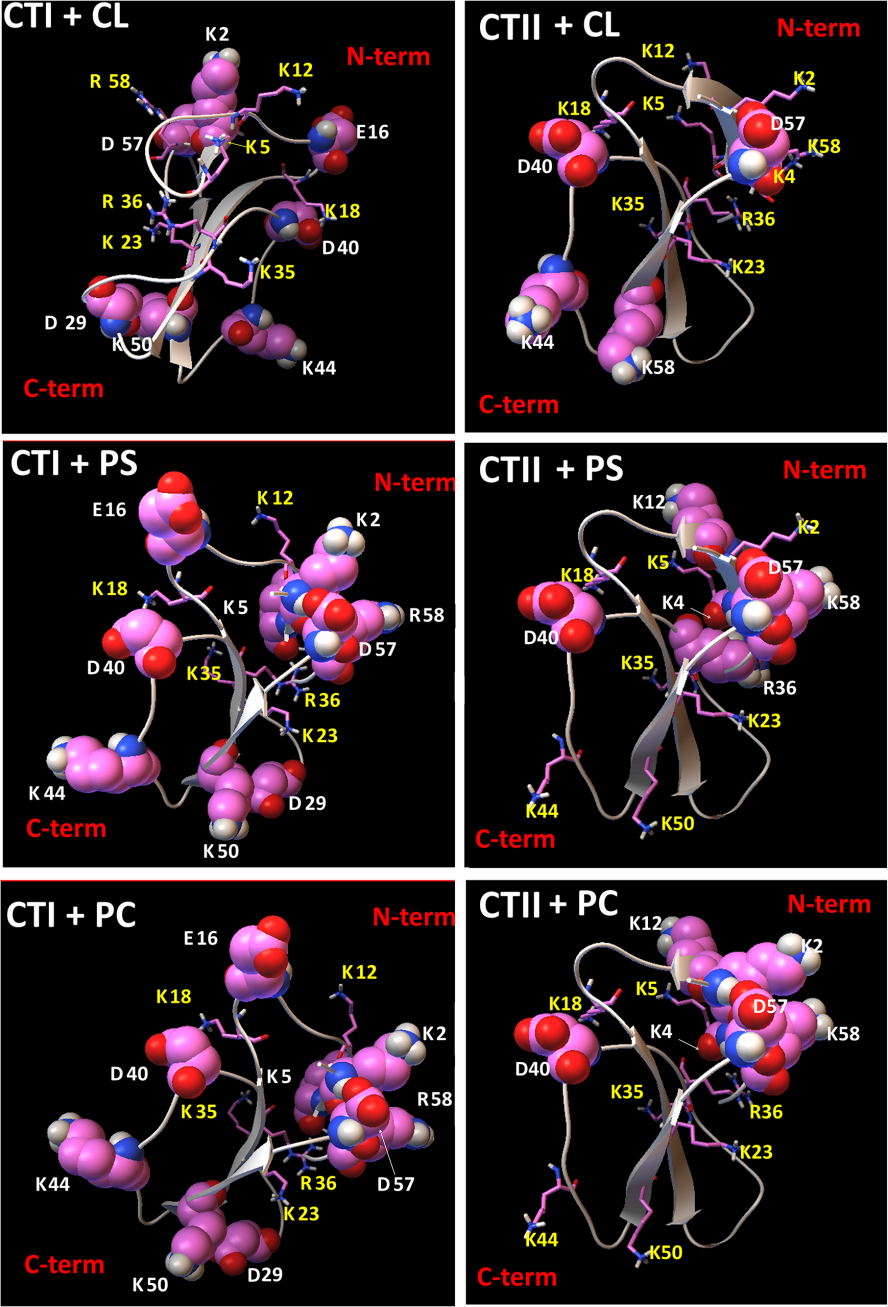
Visual summary of residues in cytotoxins that interact and do not interact with lipids. Ribbon diagrams of CTI and CTII depicting the charged amino residues (ball and stick representations) that are predicted to interact with phospholipid head groups CL (top two panels), with PS (middle panels) or with PC (bottom two panels) based on the top ranked docking conformations identified by AutoDock simulations. Interacting charged amino acid residues are marked in yellow symbols and numbers and represented in stick mode. Charged amino acid residues in CTI and CTII that do not interact with PC, PS or CL are depicted in sphere mode representations and marked in white symbols and numbers. For a complete list of interactive residues and type of bonds produced with chemical groups of lipids. (S1, S2, S3, S4, S5, S6, S7, S8, and S9 Tables).

In brief, single molecular docking analysis of nine top ranked putative docked structures for each cytotoxin suggest that the presumed PC and PS binding sites in CTI share five interacting cationic amino acid residues including K12, K18, K23, K35, R36 (S1 Table). In addition, we observed that five out of seven basic CL-interacting residues in CTI were shared in the binding sites for PC and PS. These results suggest that the interacting residues in the PC and PS biding sites in CTI are highly conserved. On the other hand, we observed K5 and R58 in CTI exclusively interact with CL (S1 Table and Fig 6).

Like CTI, the ionic residues of CTII in the putative binding sites of PC and PS are also conserved with six out of seven basic interacting amino acid residues found in PC and PS binding sites: K5, K18, K23, K35, K44, K50. Interestingly, R36 and K2 in CTII were found exclusively in PC and PS binding sites respectively. For CTI, R36 was shared in PC and PS binding sites whereas K2 is a non-interactive residue (S1 Table and Fig 6). For all top ranked docking conformations, we identified up to ten interacting basic amino acid residues in CTII that interact with CL, seven of which were shared with the binding sites of PC and PS. Hence, amino acid residues in CTII but not in CTI that exclusively interacted with CL are K4, K2,(S1 Table and Fig 6) suggesting that CTII contain a higher number of total basic residues that interact with CL compared to CTI. The presumed binding sites in CTI and CTII for PC, PS and CL also included several polar residues, which interacted with the phospholipids head groups by forming ion-polar, ion-hydrogen and hydrogen interactions (S4, S5, S6, S7, S8, and S9 Tables).

It is worth noting that most non-interacting amino acid residues for the lipid binding sites of CL and PS were located at the C-terminal region of both cytotoxins (S1 Table and Fig 6). It is conceivable that these non-interacting amino acid residues may facilitate inter-membrane contacts and lipid exchange and possibly membrane fusion.

Given that our molecular docking data suggest that PS binds to cytotoxins with stronger affinity than PC (S4, S5, S7 and S8 Tables), it is unlikely that PC molecules bind to cytotoxins in PC+PS membranes. Likewise, unlike in PC+PS liposomes, our ^1^H-NMR studies in PC+ CL liposomes and docking data (S2, S3, S4, S6, S7 and S9 Tables) suggest that CTI and CTII may bind to both PC and CL molecules in PC+CL large unilamellar membranes by binding to distinct lipid-biding sites.

In summary, our molecular docking data suggest that several basic amino acid residues of CTI and CTII, predominantly located at their N-terminal region, are involved in binding to the phospholipid head groups of CLs in the OMM whereas a different set of interacting basic amino acid residues are required to interact with PS and PC.

## Discussion

### Cytotoxins display a panoply of membrane binding and disrupting abilities

**Fig 7:**
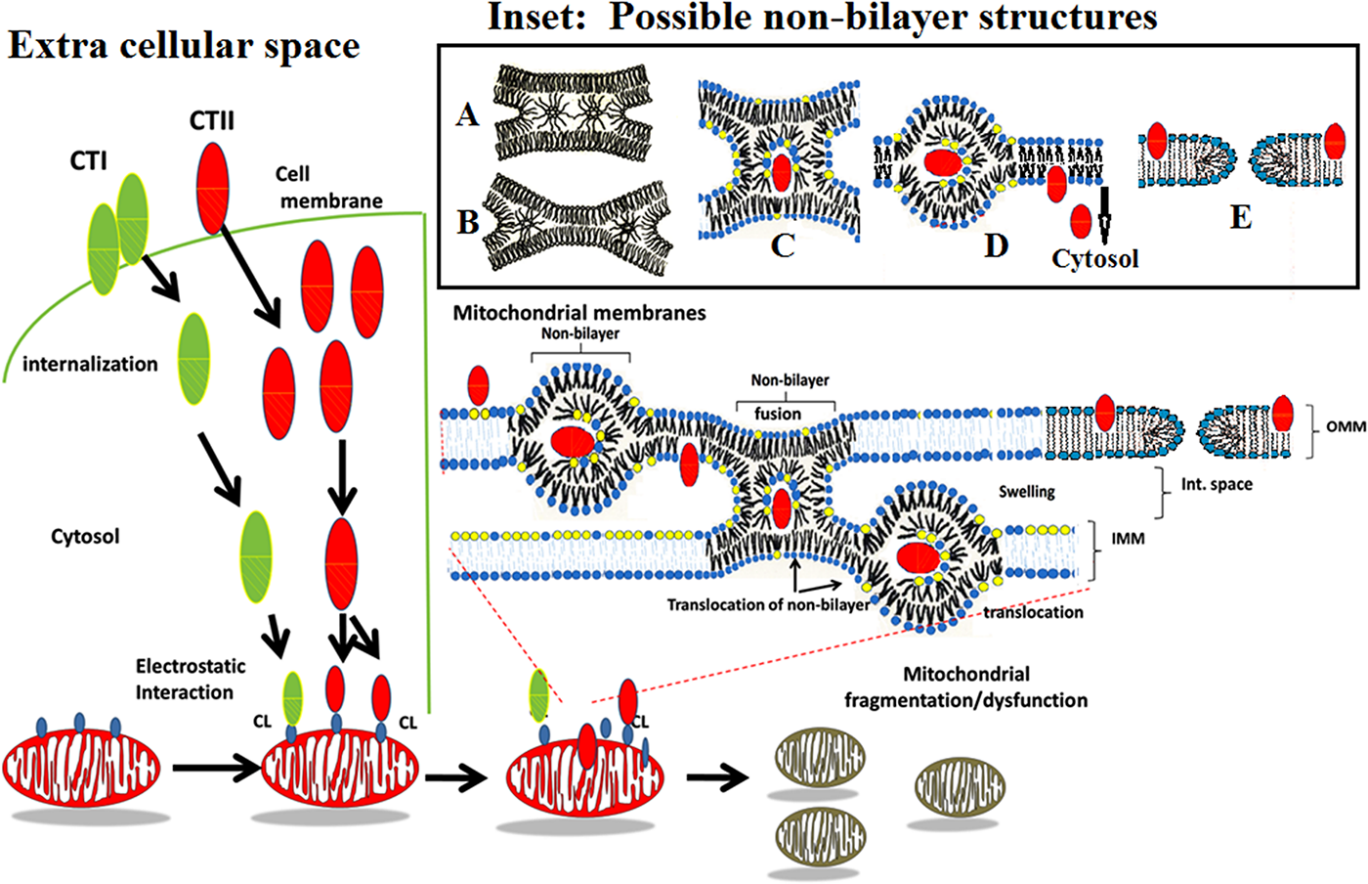
Proposed model describing the interaction of CTI and CTII with cell and mitochondrial membranes. Cytotoxins interacting with cell plasma membranes induce a contact of dehydrated surfaces of affected cells, which may result in the formation of various types of non-bilayer structures as shown in Inset: **A**—Phospholipids at a dehydrated contact zone which are not bound to a cytotoxin form transient inverted micelles responsible for ^31^P-NMR signal A shown in Fig 2 which freely exchange with a lamellar phase. The movement of these inverted micelles to the periphery of a dehydrated contact results in the formation of a trilamellar structure (**B**), an intermediate stage that precedes membrane fusion. **C** Phospholipids at a dehydrated contact which are bound to a cytotoxin may facilitate the formation of stable inverted micelle with a cytotoxin in its center. Such cytotoxin-immobilized phospholipids do not exchange with a lamellar phase within a saturation time of 0.5 s and are responsible for ^31^P-NMR signal B (Fig 2). Following membrane fusion of neighboring cells, such an inverted micelle settles in a single membrane (**D**). Due to the high surface curvature, an inverted micelle eventually undergoes a lamellar phase with the eventual release of cytotoxin into the cytosol (left side of model). In addition, due to the insertion of cytotoxins in a membrane to the depth of one monolayer [57], cytotoxins induce an asymmetric enlargement of the membrane monolayer surfaces [56] which may trigger formation of toroidal like pores (**E** (54)) which may account for ^31^P-NMR signal A (Fig 2) and the increased membrane permeability (Fig 4). Once in the cytosol, cytotoxin translocates to the mitochondrion (right side of model). Upon binding to select few CL molecules on the OMM surface, cytotoxins, sequestered by inverted micelles may translocate into the inter-membrane space where cytotoxins may further promote the fusion between the inner leaflet of an OMM and the outer leaflet of an IMM. Such molecular events are predicted to disrupt mitochondrial integrity. Phospholipid head groups of CLs are colored yellow whereas phospholipid head groups of PCs are colored blue. The hydrophobic region of CTI (green oval) and CTII (red oval) is depicted with slanted lines.

Since the isolation and purification of CTII from *Naja naja oxiana* cobra venom in 1974 [19], numerous studies over the past 40 years have offered valuable insight into the biophysical effects of CTI and CTII on cell and model membranes and characterized their cytopathological activities [4–7, 20, 22–24, 48–50]. Several research groups including ours [4–7, 20, 22–24, 48–50] and others [2, 8, 13, 14, 29, 30, 47, 51–53] have focused on elucidating the biophysical mechanisms that facilitate cytotoxin-lipid interactions. Prior to this study, we showed that these cytotoxins can permeabilize and fuse small sonicated liposomes containing high amounts of anionic phospholipids [5, 48]. Similar effects of CTI and CTII for permeabilizing and fusing of large unilamellar liposomes/lipid films containing CL or PS were observed in this study as well (Figs 4 and 5, S1, and S2 Fig). However, unlike small sonicated liposomes treated with cytotoxins, we show for the first time that CTI and CTII can promote the formation of non-bilayer structures containing immobilized phospholipids in isolated mitochondria and in large unilamellar liposomes and lipid films containing CL or PS (Fig 2), an experimental system that is considered a superior membrane model mirroring the biophysical properties of biologically-relevant lipid bilayers [24]. Collectively, our results obtained in large unilamellar liposomes, mitochondrial fractions and our molecular docking data suggest that CL is a molecular target of cytotoxins in mitochondrial membranes (Fig 2B and 2C and Fig 6) and provide a new model regarding the molecular mechanisms of these interactions (Fig 7).

### Proposed molecular mechanism by which cytotoxins disrupt mitochondrial membrane

Snake venom cytotoxins were once thought to exclusively disrupt cell membranes. However, emerging evidence suggests that snake venom cytotoxins can target mitochondria and lysosomes to promote mitochondrial dysfunction, swelling/fragmentation of mitochondria, and lysosomal rupture [8–11, 12, 31]. Furthermore, one study showed that cytotoxins can colocalize with mitochondria of intoxicated cells [10]. For the first time, we provide evidence that CTI and CTII directly associate with mitochondria and alter the molecular packing of mitochondrial phospholipids by generating two types of non-bilayer phases: one with rapid isotropic molecular mobility of phospholipids which exchange with a lamellar phase (Fig 2_,_^31^P-NMR signal A) and another with restricted isotropic mobility of phospholipids which do not exchange with a lamellar phase (Fig 2, ^31^P-NMR signal B). These non-bilayer phospholipids with rapid isotropic mobility, which exchange with a lamellar phase, are likely derived from transient inverted micelles formed at dehydrated contacts of lipid bilayers and/or from cytotoxin-induced toroidal pores [54]. Other types of non-bilayer lipids, such as normal micelles (which exist in solution) and bicontinuous cubic phase do not freely exchange with a lamellar phase, while lipids of inverted micelles at dehydrated contacts have rapid isotropic mobility and freely exchange with a lamellar phase [4, 6, 7, 55] as well as lipids at the curved toroidal pore surface [54]. Phospholipids with restricted isotropic mobility that do not exchange with a lamellar phase most likely directly interact with the cytotoxin's ionic and polar groups, which facilitates the insertion of the cytotoxin into membrane and triggers formation of a non-bilayer phase responsible for ^31^P-NMR signal B [6, 7].

Similarly, we also observed the formation of these two types of non-bilayer structures in cytotoxin-treated large unilamellar PC liposomes containing 10 mol% of either CL, the most abundant anionic phospholipid in mitochondrial membranes [16], or PS, a major anionic phospholipid in eukaryotic cells [15] that is also found in mitochondrial membranes, albeit in smaller quantities [18, 37]. CTII was able to induce formation of these two types of non-bilayer structures in both PC+CL and PC+PS large unilamellar liposomes, while CTI was able to do the same only in PC+CL large unilamellar liposomes (Fig 2D–2H).

In addition, our ^1^H-NMR permeabilization data (Fig 4) suggest that CTI and CTII may form toroidal pores [54]. Toroidal pores can form as a result of cytotoxin-induced asymmetric enlargement of the monolayer surfaces of liposomes [56]. At the same time, PC+CL liposomes treated with either CTI or CTII produced a new high-field ^1^H-NMR signal (Fig 4A and 4B) which is likely caused from a close distance between Fe(CN)_6_^−3^ ions and the N^+^(CH_3_)_3_ groups of PC [4, 6, 7, 22, 23]. This close interaction of Fe(CN)_6_^−3^ ions with the N^+^(CH_3_)_3_ groups of PC may occur in the hydrophilic core of non-bilayer structures which likely account for ^31^P-NMR signal B. This second ^31^P-NMR signal is produced when both CL and PC molecules are immobilized via the direct interaction with cytotoxins. In these non-bilayer structures, PC molecules may bind to cytotoxins via various types of polar interactions (S4 and S7 Tables). It should be noted that we did not observed a rise of a new high-field ^1^H-NMR signal (Fig 4C and 4D) in PC+PS liposomes treated with either CTI or CTII whereas ^31^P-NMR spectra of the same liposomes treated with CTII produced a non-bilayer signal B (Fig 2E). This result suggest that non-bilayer phospholipids in PC+PS liposomes that directly interact with CTII include only PS molecules, which unlike PC molecules, do not have a N^+^(CH_3_)_3_ group. This observation agrees with our docking data (S3 Table). These data suggest that PS molecules compete with PC molecules for similar binding sites on the molecular surface of CTII and have a higher binding affinity than PC.

In addition, the ^31^P NMR signals raise the possibility that CTI and CTII may be dimerizing in liposomal membranes at high concentrations. From integral intensities of ^31^P NMR signals B, which remained after applying the DANTE saturation pulses (Fig 2F–2H), we estimated the amounts of cytotoxin-immobilized phospholipids. We observed that amounts of immobilized phospholipids were not increasing proportionally with increasing toxin concentrations while the moles of immobilized lipids decreased per mole of toxin. This phenomenon could be attributed to the possibility that the phospholipid membranes were saturated at the higher concentrations of cytotoxins, leading to a slow increase in the total amount of immobilized phospholipids. On the other hand, it is conceivable that dimerization of toxin on liposomal surface was taking place, which resulted in less phospholipids binding to dimers per mole of toxin as previously suggested by previous study of cobra venom cardiotoxin A3 oligomerization in model membranes [42]. Although, previous X-ray small angle scattering studies did not detect the formation of CTII dimers in phospholipid membrane films of PC+10 mol% phosphatidic acid (PA) [57], it is possible that in liposome systems containing CL and PS, which have multiple charge centers, dimerization of CTII and CTI may take place at higher toxin concentrations. Additional studies are required to elucidate whether dimerization takes place in our system.

### Proposed mechanisms by which CTI and CTII translocate from cell membrane to mitochondria

Cytotoxins first interact with the cell membrane prior to their cytosolic translocation and interaction with mitochondria. Our data in model membranes suggest that cytotoxin-mediated destabilization of membrane bilayers and membrane surface dehydration (Figs 2 and 3 and S1 Fig) may lead to the following structural alterations in lipid membranes: 1) the formation of inverted micelles at zones of dehydrated intermembrane contacts (Fig 7, model in inset A and B) according to mechanisms we have previously described [6, 22, 24, 45] and/or 2) the formation of toroidal pore structures (Fig 7, model in inset E) resulting from the cytotoxin-induced asymmetric enlargement of monolayer surfaces of the plasma membrane [54, 56]. Phospholipids that directly interact with cytotoxins include phase segregated anionic phospholipids such as PS found in the plasma cell membrane. Cytotoxin-dehydrated PS molecules acquire a reverse wedge molecular shape which covers less area at the surface of their polar heads compared to their alkyl chains. This molecular event likely mediates the insertion of the hydrophobic core of a cytotoxin into the lipid bilayer followed by the enclosure of the cytotoxin in the center of an inverted micelle that is formed at dehydrated contact sites of neighboring cell membranes (Fig 7, model in inset C). Therefore, the formation of non-bilayer "lipidic particles" at the contact zones of neighboring membranes, which were previously described as inverted micellar inter-bilayer structures, likely promotes the fusion of cell membranes [58]. The mobility of phospholipids in inverted micelles is restricted through the direct interaction with the cytotoxin that gives rise to ^31^P-NMR signal B (Fig 2E). Following membrane fusion of neighboring membranes, inverted micelles settle in a single membrane (Fig 7, model in inset D). Due to the high surface curvature, inverted micelles eventually transform into a lamellar phase with the eventual release of cytotoxin into the cytosol. It should be noted that only CTII, but not CTI, was able to modify the bilayer packing of PS-containing membranes. This is likely due to the low hydrophobicity of loop II and smaller overall basic charge (Fig 1) which prevents CTI from efficiently penetrating into a PC+PS membrane compared to CTII. On the other hand, CTII may enable angled wedge dehydrated PS molecules, a molecular event necessary for the formation of non-bilayer structures. This concept agrees with the differences in decreased membrane disrupting activities of CTI towards membranes containing PS, which is probably due to the presence of Ser28 *in lieu* of Pro30 that is characteristic of S-type cytotoxins. Hence, the enhanced hydrophobicity and less flexible loop II is associated with increased cell membrane lytic activities of P-type cytotoxins as previously supported by experimental and computational biology data [13].

Once in the cytosol, cytotoxins are free to target mitochondria by interacting with CL molecules at the OMM. Our molecular docking data suggest that cytotoxins may initially bind to CL through CL binding sites proposed (S1 and S4 Tables), which dehydrates CL head groups and results in a reverse wedge due to the four alkyl chains of CL. Although we recognize that our docking data do not account for fact that phospholipid head groups are imbedded inside the lipid bilayer and may be sterically impeded from interacting with cytotoxins, it is conceivable that the initial electrostatic interaction of CTI or CTII with CL in the lipid bilayer may cause the lipid head groups from being partially “dislodged” from the membrane to enhance the probability of binding to cytotoxins. The three-fingered loops can enhance the interaction of CTI and CTII with CL by penetrating the lipid bilayer in an analogous manner as loop II with PS-containing membranes. In any case, dehydration of polar heads of CL greatly destabilizes the bilayer and facilitates the entry of cytotoxins' into the lipid membrane where the phosphate and glycerol groups of PC molecules are free to engage with cationic and polar residues of cytotoxins (S4 and S7 Tables). Upon gaining entry into the OMM, cytotoxins' non-interacting charged/polar residues outside lipid binding sites (S1 Table and Fig 6), which are predominantly located at the C-terminal region, may interact with polar heads from phospholipids of neighboring OMM to produce an extended dehydrated intermembrane contact region which may promote membrane fusion. This molecular event spontaneously generates an inverted micelle which forms a lipid “bridge” between neighboring membranes [55] that harbors a cytotoxin in its core (Fig 7, model in inset C). PC molecules are also drawn into this inverted micelle. These PC molecules, which adopt a cylindrical molecular shape [16], are probably drawn inside these inverted micelles to minimize the extreme curvature of a CL-generated internal micelle surface, which would have not occurred had only CL molecules been involved. Such inverted micelles which contain phospholipids immobilized by a cytotoxin agrees with our ^31^P-NMR signal B and previous studies in PC+CL membranes. In brief, these studies suggest that it takes approximately 10 to 12 lipids to cover charged and polar surface areas of CTII [6] in mitochondrial membranes and in PC+CL liposomes. Inverted micelles containing cytotoxins in their core regions may eventually mix into a lamellar phase of the OMM causing the eventual release of cytotoxins into the inter-membrane space. Although we do not have direct experimental evidence for this translocation, the observation that CTI and CTII can immobilize a significant percent of total mitochondrial phospholipids (14.3% and 19.7% respectfully), most of which is likely CL at a higher concentration than what is normally found in the OMM (3–4%), suggest that cytotoxins may translocate to the IMM. Such molecular events may not only destroy mitochondrial membrane integrity, but may also contribute to rapid mitochondrial fragmentation/swelling and dysfunction.

The enhanced cytotoxicity of CTII relative to CTI was previously correlated with the higher affinity of CTII for anionic phospholipids in cell membranes compared to CTI [13, 14]. These observations agree with our data observed in PC+PS large unilamellar membranes (Figs 2E, 3C and 3D). However, CTII and CTI exhibit similar biophysical effects on model membranes composed of PC+CL for forming non-bilayer structures, which agree with their ability to alter the lipid organization of mitochondrial membranes. We propose a conceptual model that suggests that CTII and CTI associate with CL on the OMM (Fig 7). Although both cytotoxins show high amino acid sequence conservation, our molecular docking data suggest that other N-terminally located basic residues may confer specificity for CL and not PS or PC.

Finally, our collective data may have implications for other highly basic three fingered cytotoxins. It is plausible that a similar mechanism in other cytotoxins may allow them to interact with mitochondria as suggested by the high amino acid sequence conservation of conserved putative CL-interacting amino acids (K12 and R58) across cytotoxins from different cobra species. Moreover, the biologically active loops I and III are highly conserved (Fig 1, 42% amino acid homology for loop II vs. 50% and 100% for loops I and III). However, although more knowledge has been gained regarding the molecular mechanisms that lead to cell membrane disrupting activities in P vs. S-type cytotoxins, we recognize that additional studies are required to understand the molecular basis, or identify the molecular regions, by which cytotoxins bind to CL in mitochondrial membranes.

## Supporting Information

S1 Fig CTI and CTII dehydrate phospholipid membranes containing CL. ^2^H-NMR spectra derived from ^2^H_2_O bound to the surface of membranes composed of PC+10 mol% CL (**A**), PC+10 mol% CL + CTI (**B**), PC+10 mol% CL + CTII (**C**), PC+10 mol%PS (**D**), PC+10 mol%PS+ CTI (**E**), and PC+10 mol% PS+ CTII (**F**). Molar ratio of ^2^H_2_O: lipid: cytotoxin = 10:1:100. This figure shows representative ^2^H-NMR spectra from three independent experiments that showed similar results. Each sample (**A-F**) was measured in triplicate.
(TIF)

S2 Fig Minimum amount of anionic phospholipids required to interact with CTII and CTI in phospholipid membranes. Compiled graph showing the lifetimes of erythrosine phosphorescence quenching by ferrocene in response to different molar percentage concentrations of CL and PS in PC liposomes treated with CTII or CTI. τn and τo denote the lifetimes of phosphorescence in the presence and absence of CTII or CTI respectively at a cytotoxin to lipid molar ratio of 0.01. The graph shows compiled means and standard errors from three independent erythrosine phosphorescence experiments (*:p<0.05 of untreated PC +CL/ PC + PS vs. respective PC +CL/ PC + PS treated with CTI or CTII).
(TIF)

S1 Table Summary of interactive and non-interactive residues in the lipid binding sites of CTI and CTII. Ionic amino acid residues in cytotoxins CTI and CTII inside and outside hypothetical binding sites of phosphatidylcholine (PC), phosphatidylserine (PS) and cardiolipin (CL) as determined by AutoDock modeling.
(DOCX)

S2 Table Summary of amino acid residues in CTI that are shared among lipid binding sites. Amino acid residues on the molecular surface of CTI that interact with the phospholipid head groups of CL, PC, and PS based on a total of nine top ranking docked conformations as determined by AutoDock. In addition, the table also shows the total number of amino acids that interact with either PS or CL and that are shared with PC binding sites. For the complete list of amino acid residues that interact with CTI (S5 and S6 Tables). Numbers in columns (№) correspond to the binding site number in the order by which it was ranked by AutoDock. Energies of binding affinities are expressed in kcal/mol. The subscript _pb_ denotes a peptide bond. NA denotes not applicable.
(DOCX)

S3 Table Summary of amino acid residues in CTII that are shared among lipid binding sites. Amino acid residues on the molecular surface of CTII that bind to the phospholipid head group of CL, PC, and PS based on a total of nine top ranking docked conformations as determined by AutoDock. In addition, the table also shows the total number of amino acids that interact with either PS or CL and that are shared with PC binding sites. For the complete list of amino acid residues that interact with CTII (S8 and S9 Tables). Numbers in columns (№) correspond to the binding site number in the order by which it was ranked by AutoDock. Energies of binding affinities are expressed in kcal/mol. Subscript _pb_ denotes peptide bonds. NA: Not applicable NA denotes not applicable.
(DOCX)

S4 Table Summary of residues in CTI that bind to PC. Hypothetical binding sites in CTI that bind to the phospholipid head group of PC as determined by AutoDock modeling. The table shows the complete list of amino acid residues in CTI that interact with the charged and polar groups of PC at various binding sites. Pb in C = O_pb_^σ−^ or in NH_pb_^σ+^ means peptide bond. NA denotes not applicable.
(DOCX)

S5 Table Summary of amino acid residues in CTI that interact with PS. Hypothetical binding sites in CTI that bind to the phospholipid head group of PS as determined by AutoDock modeling. The table shows a complete list of amino acid residues in CTI that interact with the charged and polar groups of PS for various binding sites. Pb in C = O_pb_^σ−^ or in NH_pb_^σ+^ denotes a peptide bond.
(DOCX)

S6 Table Summary of amino acid residues in CTI that interact with CL. Hypothetical binding sites in CTI that bind to the phospholipid head group of CL as determined by AutoDock modeling. The table shows a complete list of amino acid residues in CTI that interact with the CL charged and polar groups at various binding sites. Pb in C = O_pb_^σ−^ or in NH_pb_^σ+^ denotes a peptide bond.
(DOCX)

S7 Table Summary of amino acid residues in CTII that interact with PC. Hypothetical binding sites in CTII that bind to the phospholipid head group of PC as determined by AutoDock modeling. The table shows a complete list of amino acid residues in CTII that interact with the charged and polar groups of PC at various binding sites. Pb in C = O_pb_^σ−^ or in NH_pb_^σ+^ denotes a peptide bond.
(DOCX)

S8 Table Summary of amino acid residues in CTII that interact with PS. Hypothetical binding sites in CTII that bind to the phospholipid head group of PS as determined by AutoDock modeling. The table shows a complete list of amino acid residues in CTII that interact with the charged and polar groups of PS for various binding sites. Pb in C = O_pb_^σ−^ or in NH_pb_^σ+^ denotes a peptide bond.
(DOCX)

S9 Table Summary of amino acid residues in CTII that interact with CL. Hypothetical binding sites in CTII that bind to the phospholipid head group of CL as determined by AutoDock modeling. The table shows a complete list of amino acid residues in CTII that interact with the charged and polar groups of CL at various binding sites. Pb in C = O_pb_^σ−^ or in NH_pb_^σ+^ denotes a peptide bond.
(DOCX)
