# Supplementary figures and images for: Naja naja oxiana Cobra Venom Cytotoxins CTI and CTII Disrupt Mitochondrial Membrane Integrity: Implications for Basic Three-Fingered Cytotoxins
Source: PLoS One. 2015 Jun 19;10(6):e0129248. doi: 10.1371/journal.pone.0129248 (PMC4474699; doi:10.1371/journal.pone.0129248)

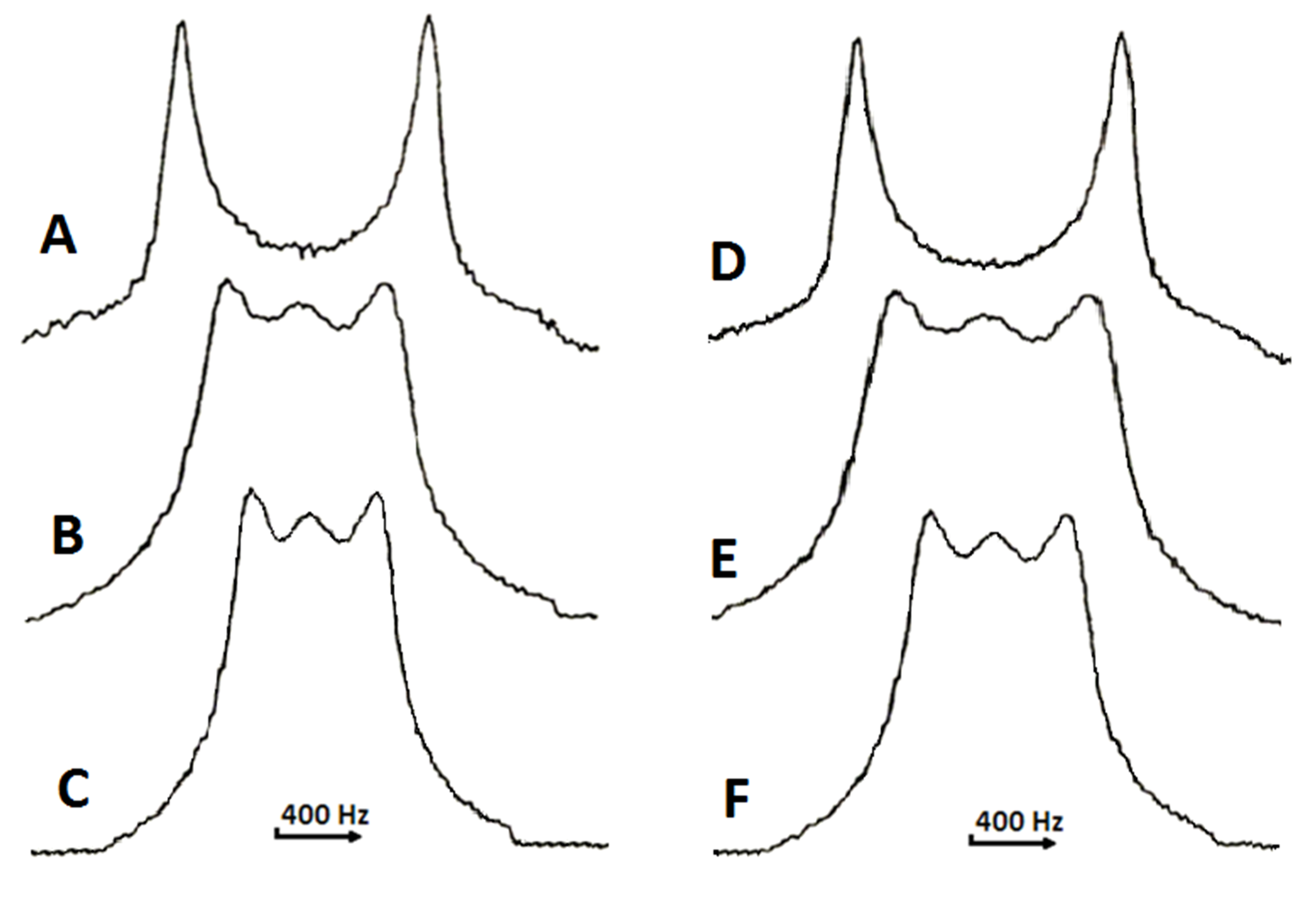

Supplement: S1 Fig — 2H-NMR spectra derived from 2H2O bound to the surface of membranes composed of PC+10 mol% CL (A), PC+10 mol% CL + CTI (B), PC+10 mol% CL + CTII (C), PC+10 mol%PS (D), PC+10 mol%PS+ CTI (E), and PC+10 mol% PS+ CTII (F). Molar ratio of 2H2O: lipid: cytotoxin = 10:1:100. This figure shows representative 2H-NMR spectra from three independent experiments that showed similar results. Each sample (A-F) was measured in triplicate. (TIF) [file pone.0129248.s001.tif]

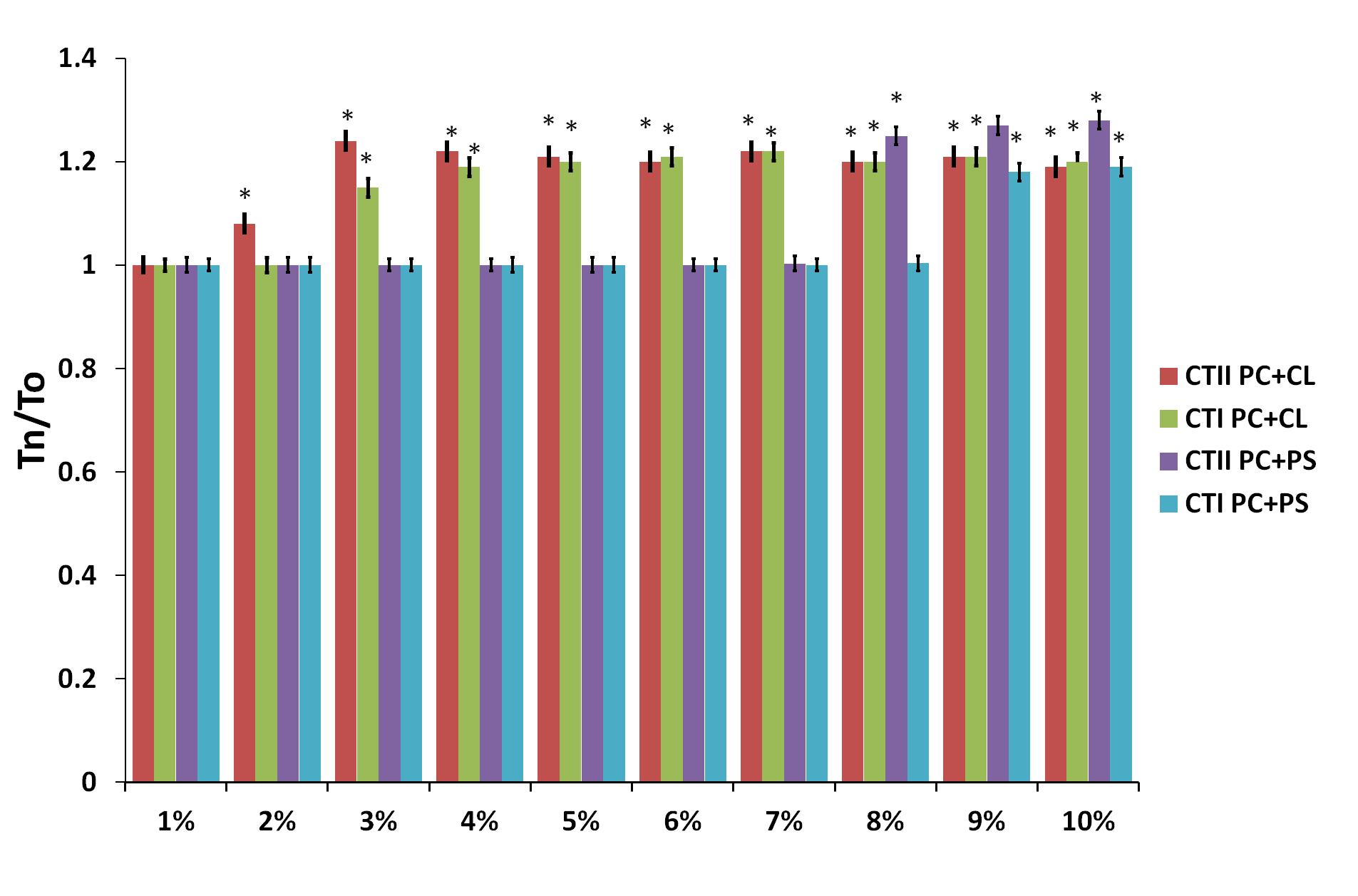

Supplement: S2 Fig — Compiled graph showing the lifetimes of erythrosine phosphorescence quenching by ferrocene in response to different molar percentage concentrations of CL and PS in PC liposomes treated with CTII or CTI. τn and τo denote the lifetimes of phosphorescence in the presence and absence of CTII or CTI respectively at a cytotoxin to lipid molar ratio of 0.01. The graph shows compiled means and standard errors from three independent erythrosine phosphorescence experiments (*:p<0.05 of untreated PC +CL/ PC + PS vs. respective PC +CL/ PC + PS treated with CTI or CTII). (TIF) [file pone.0129248.s002.tif]
