# Supplementary material for: Naja naja oxiana Cobra Venom Cytotoxins CTI and CTII Disrupt Mitochondrial Membrane Integrity: Implications for Basic Three-Fingered Cytotoxins
Source: PLoS One. 2015 Jun 19;10(6):e0129248. doi: 10.1371/journal.pone.0129248 (PMC4474699; doi:10.1371/journal.pone.0129248)
Supplement: S1 Table — Ionic amino acid residues in cytotoxins CTI and CTII inside and outside hypothetical binding sites of phosphatidylcholine (PC), phosphatidylserine (PS) and cardiolipin (CL) as determined by AutoDock modeling. (DOCX) [file pone.0129248.s003.docx]

**S1 Table. Summary of interactive and non-interactive residues in the lipid binding sites of CTI and CTII**

| Cyto-toxin | **Ionic residues of cytotoxin that interact with PC** | | **Ionic residues of cytotoxin that interact with PS** | | **Ionic residues of cytotoxin that interact with CL** | |
| --- | --- | --- | --- | --- | --- | --- |
|  | Within binding sites | outside binding sites | within binding sites | outside binding sites | within binding sites | outside binding sites |
| **CTI** | K^+^12, K^+^18, K^+^23, K^+^35, R^+^36 | K^+^2, K^+^5, E^‒^16, D^‒^29, D^‒^40, K^+^44, K^+^50, D^‒^57, R^+^58 | K^+^12, K^+^18, K^+^23, K^+^35, R^+^36 | K^+^2, K^+^5, E^‒^16, D^‒^29, D^‒^40,  K^+^44, K^+^50, D^‒^57, R^+^58 | K^+^5, K^+^12, K^+^18, K^+^23, K^+^35, R^+^36, R^+^58 | K^+^2, E^‒^16, D^‒^29, D^‒^40, K^+^44, K^+^50, D^‒^57 |
| **CTII** | K^+^5, K^+^18, K^+^23, K^+^35, R^+^36, K^+^44, K^+^50 | K^+^2, K^+^4, K^+^12, H^+^31, D^‒^40, D^‒^57, R^+^58 | K^+^2, K^+^5, K^+^18, K^+^23, K^+^35, K^+^44, K^+^50 | K^+^4, K^+^12, H^+^31, R^+^36, D^‒^40, D^‒^57, R^+^58 | K^+^2, K^+^4, K^+^5, K^+^12, K^+^18, K^+^23, H^+^31, K^+^35, R^+^36, R^+^58 | D^‒^40, K^+^44, K^+^50, D^‒^57 |

Ionic amino acid residues in cytotoxins CTI and CTII inside and outside hypothetical binding sites of phosphatidylcholine (PC), phosphatidylserine (PS) and cardiolipin (CL) as determined by AutoDock modeling.
