# Supplementary material for: Naja naja oxiana Cobra Venom Cytotoxins CTI and CTII Disrupt Mitochondrial Membrane Integrity: Implications for Basic Three-Fingered Cytotoxins
Source: PLoS One. 2015 Jun 19;10(6):e0129248. doi: 10.1371/journal.pone.0129248 (PMC4474699; doi:10.1371/journal.pone.0129248)
Supplement: S2 Table — Amino acid residues on the molecular surface of CTI that interact with the phospholipid head groups of CL, PC, and PS based on a total of nine top ranking docked conformations as determined by AutoDock. In addition, the table also shows the total number of amino acids that interact with either PS or CL and that are shared with PC binding sites. For the complete list of amino acid residues that interact with CTI (S5 and S6 Tables). Numbers in columns (№) correspond to the binding site number in the order by which it was ranked by AutoDock. Energies of binding affinities are expressed in kcal/mol. The subscript pb denotes a peptide bond. NA denotes not applicable. (DOCX) [file pone.0129248.s004.docx]

| **Amino acid residues in CTI that interact with the phospholipid headgroup of PC** | | | **Interactive residues in CTI that are shared between the phospholipid head group binding sites of PS and PC (shown are CT residues docked with PS)** | | | **Interactive residues in CTI that are shared between the phospholipid head group binding sites of CL and PC**  **(shown are CT residues docked with CL)** | | |
| --- | --- | --- | --- | --- | --- | --- | --- | --- |
| № | Affinity  (kcal/mol) | Interactive residues | № | Affinity  (kcal/mol) | Interactive residues | № | Affinity  (kcal/mol) | Interactive residues |
| 1 | -3.5 | **K^+^12**,**K12**(NH_pb_ ^σ+^),**K^+^35**,**R36**(C=O_pb_^σ−^),**C38**(NH_pb_^σ+^) | 1 | -3.7 | **K^+^12**, **K^+^35**, **R36**(C=O_pb_^σ−^),**C38**(NH_pb_^σ+^) | 1 | -4.7 | **K^+^12**,**K12**(NH_pb_ ^σ+^),**K^+^35**,**R36**(C=O_pb_^σ−^),**C38**(NH_pb_^σ+^) |
| 2 | -3.3 | **L6**(NH_pb_^σ+^),**K12**(NH_pb_^σ+^),**K^+^18**, **K^+^35**, **C38**(NH_pb_^σ+^) | 1 | -3.7 | **L6**(NH_pb_^σ+^),**K12**(NH_pb_^σ+^),**K^+^35**,**C38**(NH_pb_^σ+^) | 1 | -4.7 | **L6**(NH_pb_^σ+^),**K12**(NH_pb_^σ+^),**K^+^18**, **K^+^35**, **C38**(NH_pb_^σ+^) |
| 3 | -3.0 | **K^+^23**, **R^+^36** | 2 | -3.7 | **K^+^23**, **R^+^36** | 9 | -3.9 | **R^+^36** |
| 4 | -2.7 | **S46**(OH^σ+^), **Y51**(OH^σ+^), **Y22**(OH^σ+^) | 1 | -3.7 | **Y22**(OH^σ+^) | NA | NA | **no match** |
| 5 | -2.6 | **K^+^23**, **C42**(NH_pb_^σ+^), **C54**(NH_pb_ ^σ+^) | 2 | -3.7 | **K^+^23** | NA | NA | **no match** |
| 6 | -2.5 | **K^+^12**, **K^+^18, Y22**(OH^σ+^), **K^+^35** | 1 | -3.7 | **K^+^12**, **K^+^18, Y22**(OH^σ+^), **K^+^35** | 1 | -4.7 | **K^+^12**, **K^+^18, K^+^35** |
| 7 | -2.4 | **G17**(NH_pb_^σ+^), **N19**(NH_2_^σ+^), **N19**(NH_pb_^σ+^) | NA | NA | **no match** | NA | NA | **no match** |
| 8 | -2.4 | **G17**(NH_pb_^σ+^), **G17**(NH_pb_^σ+^) | NA | NA | **no match** | NA | NA | **no match** |
| 9 | -2.4 | **R^+^36**, **N60**(COO^−^) | 2 | -3.7 | **R^+^36** | 9 | -3.9 | **R^+^36** |

**S2 Table. Summary of amino acid residues in CTI that are shared among lipid binding sites.**

Amino acid residues on the molecular surface of CTI that interact with the phospholipid head groups of CL, PC, and PS based on a total of nine top ranking docked conformations as determined by AutoDock. In addition, the table also shows the total number of amino acids that interact with either PS or CL and that are shared with PC binding sites. For the complete list of amino acid residues that interact with CTI refer to Tables S6 and S8. Numbers in columns (№) correspond to the binding site number in the order by which it was ranked by AutoDock. Energies of binding affinities are expressed in kcal/mol. The subscript _pb_ denotes a peptide bond.

NA denotes not applicable.
