# Supplementary material for: Naja naja oxiana Cobra Venom Cytotoxins CTI and CTII Disrupt Mitochondrial Membrane Integrity: Implications for Basic Three-Fingered Cytotoxins
Source: PLoS One. 2015 Jun 19;10(6):e0129248. doi: 10.1371/journal.pone.0129248 (PMC4474699; doi:10.1371/journal.pone.0129248)
Supplement: S4 Table — Hypothetical binding sites in CTI that bind to the phospholipid head group of PC as determined by AutoDock modeling. The table shows the complete list of amino acid residues in CTI that interact with the charged and polar groups of PC at various binding sites. Pb in C = Opb σ− or in NHpb σ+ means peptide bond. NA denotes not applicable. (DOCX) [file pone.0129248.s006.docx]

| Binding site # | *PC polar groups* | *CTI amino acid residues* | *Bond type and orientation* |
| --- | --- | --- | --- |
| **Binding site 1**  Affinity (kcal/mol)  ‒3.5 | **PO_4_^–^** | **C38**(NH_pb_^σ+^) | ion-hydrogen |
|  | **1CO**^σ^**^–^C** | **K^+^12**(N^+^H_3_), | ion-polar |
|  | **1C=O**^σ−^ | **K^+^12**(N^+^H_3_), | ion-polar |
|  | **2C=O**^σ−^ | **K12**(NH_pb_ ^σ+^) | hydrogen |
|  | **N^+^(CH_3_)_3_** | **K^+^35**(N^+^H_3_), **R36**(C=O_pb_^σ−^) | ionic repulsion, ion-polar |
| **Binding site 2**  Affinity (kcal/mol)  ‒3.3 | **PO_4_^–^** | **K^+^35**(N^+^H_3_), **C38**(NH_pb_^σ+^) | ion-polar, ion-hydrogen |
|  | **1C=O**^σ−^ | **K^+^35**(N^+^H_3_), **L6**(NH_pb_^σ+^) | ion-polar, hydrogen |
|  | **2** **C=O**^σ−^ | **K12**(NH_pb_^σ+^) | hydrogen |
|  | **N^+^(CH_3_)_3_** | **K^+^18**(N^+^H_3_) | ionic repulsion |
| **Binding site 3**  Affinity (kcal/mol)  ‒3.0 | **PO_4_^–^** | **R^+^36**(=N^+^H_2_) | ionic |
|  | **1CO**^σ^**^–^C** | **K^+^23**(N^+^H_3_) | ion-polar |
|  | **1C=O**^σ−^ | **K^+^23**(N^+^H_3_) | ion-polar |
|  | **2CO**^σ^**^–^C** | **R^+^36**(=N^+^H_2_) | ion-polar |
|  | **2C=O**^σ−^ | **R^+^36**(=N^+^H_2_) | ion-polar |
|  | **N^+^(CH_3_)_3_** | **None** | into solution |
| **Binding site 4**  Affinity (kcal/mol)  ‒2.7 | **PO_4_^–^** | **S46**(OH^σ+^), **Y51**(OH^σ+^) | 2 ion-hydrogen |
|  | **1C=O**^σ−^ | **Y22**(OH^σ+^) | hydrogen |
|  | **2C=O**^σ−^ | **S46**(OH^σ+^) | hydrogen |
|  | **N^+^(CH_3_)_3_** | **None** | into solution |
| **Binding site 5**  Affinity (kcal/mol)  ‒2.6 | **PO_4_^–^** | **K^+^23**(N^+^H_3_), **C54**(NH_pb_ ^σ+^) | ionic, ion-hydrogen |
|  | **1C=O**^σ−^ | **C42**(NH_pb_^σ+^) | hydrogen |
|  | **2C=O**^σ−^ | **C42**(NH_pb_^σ+^) | hydrogen |
|  | **N^+^(CH_3_)_3_** | **None** | into solution |
| **Binding site 6**  Affinity (kcal/mol)  ‒2.5 | **PO_4_^–^** | **K^+^12**(N^+^H_3_), **K^+^18**(N^+^H_3_) | 2 ionic |
|  | **1C=O**^σ−^ | **K^+^12**(N^+^H_3_), **K^+^35**(N^+^H_3_) | 2 ion-polar |
|  | **2CO**^σ^**^–^C** | **K^+^18**(N^+^H_3_) | ion-polar |
|  | **2C=O** ^σ−^ | **Y22**(OH^σ+^) | hydrogen |
|  | **N^+^(CH_3_)_3_** | **None** | into solution |
| **Binding site 7**  Affinity (kcal/mol)  ‒2.4 | **PO_4_^–^** | **N19**(NH_pb_^σ+^) | ion-hydrogen |
|  | **1C=O**^σ−^ | **N19**(NH_2_^σ+^) | hydrogen |
|  | **2CO**^σ^**^–^C** | **G17**(NH_pb_^σ+^) | hydrogen |
|  | **N^+^(CH_3_)_3_** | None | into solution |
| **Binding site 8**  Affinity (kcal/mol)  ‒2.4 | **PO_4_^–^** | **N19**(NH_pb_^σ+^) | ion-hydrogen |
|  | **1CO**^σ^**^–^C** | **G17**(NH_pb_^σ+^) | hydrogen |
|  | **2C=O**^σ−^ | **G17**(NH_pb_^σ+^) | 2 hydrogen |
|  | **N^+^(CH_3_)_3_** | **None** | into solution |
| **Binding site 9**  Affinity (kcal/mol)  ‒2.4 | **PO_4_^–^** | **None** | into solution |
|  | **1CO**^σ^**^–^C** | **R^+^36**(=N^+^H_2_) | ion-polar |
|  | **1C=O**^σ−^ | **R^+^36**(=N^+^H_2_) | ion-polar |
|  | **N^+^(CH_3_)_3_** | **N60**(COO^−^) | ionic |

**S4 Table. Summary of residues in CTI that bind to PC.**

Hypothetical binding sites in CTI that bind to the phospholipid head group of PC as determined by AutoDock modeling. The table shows the complete list of amino acid residues in CTI that interact with the PC charged and polar groups at various binding sites. Pb in C=O_pb_^σ−^ or in NH_pb_^σ+^ denotes a peptide bond
