# Supplementary material for: Naja naja oxiana Cobra Venom Cytotoxins CTI and CTII Disrupt Mitochondrial Membrane Integrity: Implications for Basic Three-Fingered Cytotoxins
Source: PLoS One. 2015 Jun 19;10(6):e0129248. doi: 10.1371/journal.pone.0129248 (PMC4474699; doi:10.1371/journal.pone.0129248)
Supplement: S5 Table — Hypothetical binding sites in CTI that bind to the phospholipid head group of PS as determined by AutoDock modeling. The table shows a complete list of amino acid residues in CTI that interact with the charged and polar groups of PS for various binding sites. Pb in C = Opb σ− or in NHpb σ+ denotes a peptide bond. (DOCX) [file pone.0129248.s007.docx]

| Binding site | *PS polar groups* | *CTI amino acid residues* | *Bond type and orientation* |
| --- | --- | --- | --- |
| **Binding site 1**  Affinity (kcal/mol)  ‒3.7 | **PO_4_^–^** | **K^+^18**(N^+^H_3_), **C38**(NH_pb_ ^σ+^), **Y22**(OH^σ+^) | ionic, 2 ion-hydrogen, i.s. |
|  | **COO^–^** | **L6**(NH_pb_ ^σ+^), **K^+^35**(N^+^H_3_) | ion-hydrogen, ionic, i.s. |
|  | **1CO**^σ^**^–^C** | **K12**(NH_pb_^σ+^) | hydrogen |
|  | **1C=O**^σ−^ | **K^+^12**(N^+^H_3_) | ion-polar |
|  | **2C=O**^σ−^ | **K^+^12**(N^+^H_3_) | ion-polar |
|  | **N^+^H_3_** | **R36**(C=O_pb_^σ−^) | ion-polar |
| **Binding site 2**  Affinity (kcal/mol)  ‒3.7 | **PO_4_^–^** | **K^+^23**(N^+^H_3_), **R^+^36**(=N^+^H_2_) | 2 ionic |
|  | **COO^–^** | **None** | into solution |
|  | **2** **C=O**^σ−^ | **R^+^36**(=N^+^H_2_) | ion-polar |
|  | **N^+^H_3_** | **D^−^29**(C=O_pb_^σ−^), **T31**(C=O_pb_^σ−^) | 2 ion-polar |
| **Binding site 3**  Affinity (kcal/mol)  ‒3.7 | **PO_4_^–^** | **C38**(NH_pb_^σ+^), **Y22**(OH ^σ+^) | 2 ion-hydrogen, into solution |
|  | **COO^–^** | **K12**(NH_pb_^σ+^) | ion-hydrogen |
|  | **1CO**^σ^**^–^C** | **K^+^35**(N^+^H_3_) | ion-polar |
|  | **1C=O** ^σ−^ | **K^+^35**(N^+^H_3_) | ion-polar |
|  | **2COC**^σ^**^–^** | **K^+^35**(N^+^H_3_) | ion-polar |
|  | **2C=O** ^σ−^ | **L6**(OH^σ+^) | hydrogen |
|  | **N^+^H_3_** | **T13**(C=O_pb_^σ−^) | ion-polar |
| **Binding site 4**  Affinity (kcal/mol)  ‒3.7 | **PO_4_^–^** | **K^+^12**(N^+^H_3_) | ionic, |
|  | **1C=O** ^σ−^ | **C38**(NH_pb_^σ+^) | hydrogen |
|  | **N^+^H_3_** | **K12**(NH_pb_ ^σ+^), **K12**(C=O_pb_^σ−^) | ion-hydrogen repulsion, ion-polar |
| **Binding site 5**  Affinity (kcal/mol)  ‒3.6 | **PO_4_^–^** | **K12**(NH_pb_ ^σ+^), **N60**(-NH_2_ ^σ+^) | 2 ion-hydrogen |
|  | **COO^–^** | **L6**(**O**H^σ+^), **K^+^35**(N^+^H_3_) | ion-hydrogen, ionic |
|  | **1C=O** ^σ−^ | **K^+^12**( N^+^H_3_) | ion-polar |
|  | **2C=O** ^σ−^ | **Y22**(OH^σ+^) | hydrogen |
|  | **N^+^H_3_** | **Y22**(O^σ−^H) | ion-polar |
| **Binding site 6**  Affinity (kcal/mol)  ‒3.6 | **PO_4_^–^** | **None** | into solution |
|  | **COO^–^** | **K12**(NH_pb_^σ+^) | ion-hydrogen |
|  | **1CO**^σ^**^–^C** | **Y22**(OH^σ+^) | hydrogen |
|  | **2CO**^σ^**^–^C** | **Y22**(OH^σ+^) | hydrogen |
|  | **2C=O** ^σ−^ | **K^+^18**(N^+^H_3_) | ion-polar |
|  | **N^+^H_3_** | **T13**(C=O_pb_^σ−^) | ion-polar |
| **Binding site 7**  Affinity (kcal/mol)  ‒3.5 | **PO_4_^–^** | **K12**(NH_pb_^σ+^), **C38**(NH_pb_^σ+^) | 2 ion-hydrogen |
|  | **COO^–^** | **L6**(**O**H^σ+^), **K^+^35**(N^+^H_3_) | ion-hydrogen, ionic |
|  | **1CO**^σ^**^–^C** | **C38**(NH_pb_^σ+^) | ion-hydrogen |
|  | **1C=O** ^σ−^ | **Y22**(OH^σ+^) | hydrogen |
|  | **2C=O** ^σ−^ | **K^+^12**(N^+^H_3_), **K12**(NH_pb_^σ+^) | ion-polar, hydrogen |
|  | **N^+^H_3_** | **R36**(C=O_pb_^σ−^) | ion-polar |
| **Binding site 8**  Affinity (kcal/mol)  ‒3.5 | **PO_4_^–^** | **Y22**(OH^σ+^) | ion-hydrogen, into solution |
|  | **COO^–^** | **L6**(**O**H^σ+^), **K^+^35**(N^+^H_3_) | ion-hydrogen, ionic |
|  | **2C=O** ^σ−^ | **C38**(NH_pb_^σ+^) | hydrogen |
|  | **N^+^H_3_** | **K^+^35**(N^+^H_3_) | ionic repulsion, into solution |
| **Binding site 9**  Affinity (kcal/mol)  ‒3.4 | **PO_4_^–^** | **K^+^5**(N^+^H_3_) | ionic, into solution |
|  | **COO^–^** | **Y22**(OH ^σ+^), **K^+^35**(N^+^H_3_) | ion-polar, ionic |
|  | **N^+^H_3_** | **R36**(C=O_pb_^σ−^) | ion-polar |

**S5 Table. Summary of amino acid residues in CTI that interact with PS.**

Hypothetical binding sites in CTI that bind to the phospholipid headgroup of PS as determined by AutoDock modeling. The table shows a complete list of amino acid residues in CTI that interact with the PS charged and polar groups for various binding sites. Pb in C=O_pb_^σ−^ or in NH_pb_^σ+^ denotes a peptide bond.
