# Supplementary material for: Naja naja oxiana Cobra Venom Cytotoxins CTI and CTII Disrupt Mitochondrial Membrane Integrity: Implications for Basic Three-Fingered Cytotoxins
Source: PLoS One. 2015 Jun 19;10(6):e0129248. doi: 10.1371/journal.pone.0129248 (PMC4474699; doi:10.1371/journal.pone.0129248)
Supplement: S6 Table — Hypothetical binding sites in CTI that bind to the phospholipid head group of CL as determined by AutoDock modeling. The table shows a complete list of amino acid residues in CTI that interact with the CL charged and polar groups at various binding sites. Pb in C = Opb σ− or in NHpb σ+ denotes a peptide bond. (DOCX) [file pone.0129248.s008.docx]

**S6 Table. Summary of amino acid residues in CTI that bind to CL.**

| Binding site # | *CL polar groups* | *CTI amino acid residues* | *Bond type and orientation* |
| --- | --- | --- | --- |
| **Binding site 1**  Affinity (kcal/mol)  ‒4.7 | **APO_4_^–^** | **C38**(NH_pb_^σ+^) | ion-hydrogen |
|  | **BPO_4_^–^** | **K^+^35**(N^+^H_3_), **R36**(NH_pb_^σ+^) | ionic, ion-hydrogen |
|  | **1CO** ^σ^**^–^** | **L6**(NH_pb_^σ+^) | hydrogen |
|  | **2C=O**^σ−^ | **K12**(NH_pb_^σ+^) | hydrogen |
|  | **3C=O**^σ−^ | **K^+^12** (N^+^H_3_) | ion-polar |
|  | **4C=O**^σ−^ | **K^+^18** (N^+^H_3_) | ion-polar |
|  | **5C=O**^σ−^ | **K^+^18** (N^+^H_3_) | ion-polar |
| **Binding site 2**  Affinity (kcal/mol)  ‒4.4 | **APO_4_^–^** | **K^+^12**(N^+^H_3_), **K12**(NH_pb_^σ+^) | ionic, ion-hydrogen |
|  | **BPO_4_^–^** | **K^+^35**(N^+^H_3_), **R36**(NH_pb_^σ+^) | ionic, ion-hydrogen |
|  | **2C=O**^σ−^ | **K^+^12**(N^+^H_3_) | ion-polar |
|  | **4C=O**^σ−^ | **K^+^18** (N^+^H_3_) | ion-polar |
|  | **5C=O**^σ−^ | **K^+^18** (N^+^H_3_) | ion-polar |
| **Binding site 3**  Affinity (kcal/mol)  ‒4.3 | **APO_4_^–^** | **K^+^35**(N^+^H_3_), **R36**(NH_pb_^σ+^) | ionic, ion-hydrogen |
|  | **BPO_4_^–^** | **None** | into solution |
|  | **1CO** ^σ^**^–^** | **K^+^35**(N^+^H_3_) | ion-polar |
|  | **3C=O**^σ−^ | **K^+^18**(N^+^H_3_) | ion-polar |
|  | **4C=O**^σ−^ | **K^+^12**(NH_pb_^σ+^) | hydrogen |
|  | **5C=O**^σ−^ | **K^+^18**(N^+^H_3_), **C38**( NH_pb_^σ+^) | ion-polar, hydrogen |
| **Binding site 4**  Affinity (kcal/mol)  ‒4.2 | **APO_4_^–^** | **None** | into solution |
|  | **BPO_4_^–^** | **K^+^35**(N^+^H_3_), **R36**(NH_pb_^σ+^) | ionic, ion-hydrogen |
|  | **1CO** ^σ^**^–^** | **K^+^35**(N^+^H_3_) | ion-polar |
|  | **4COC**^σ^**^–^** | **K12**(NH_pb_^σ+^) | hydrogen |
|  | **5COC**^σ^**^–^** | **C38**(NH_pb_^σ+^) | hydrogen |
| **Binding site 5**  Affinity (kcal/mol)  ‒4.1 | **APO_4_^–^** | **K^+^35**(N^+^H_3_), **R36**(NH_pb_^σ+^) | ionic, ion-hydrogen |
|  | **BPO_4_^–^** | **R36**(NH_pb_^σ+^) | ion-hydrogen |
|  | **1CO** ^σ^**^–^** | **R36**(NH_pb_^σ+^) | hydrogen |
|  | **2C=O**^σ−^ | **K12**(NH_pb_^σ+^) | hydrogen |
|  | **3CO**^σ^**^–^C** | **C38**(NH_pb_^σ+^) | hydrogen |
|  | **3C=O**^σ−^ | **K^+^18**(N^+^H_3_) | ion-polar |
| **Binding site 6**  Affinity (kcal/mol)  ‒4.0 | **APO_4_^–^** | **L6**(NH_pb_^σ+^), **K^+^35**(N^+^H_3_), **R36**(NH_pb_^σ+^) | ionic, 2 ion-hydrogen |
|  | **BPO_4_^–^** | **K12**(NH_pb_^σ+^) | ion-polar |
|  | **2C=O**^σ−^ | **K^+^35**(N^+^H_3_) | ion-polar |
|  | **3C=O**^σ−^ | **K^+^5** (N^+^H_3_) | ion-polar |
|  | **3CO**^σ^**^–^C** | **L6**(NH_pb_^σ+^), | hydrogen |
|  | **4C=O**^σ−^ | **K^+^12** (N^+^H_3_) | ion-polar |
|  | **5C=O**^σ−^ | **C38**(NH_pb_^σ+^) | hydrogen |
|  | **4CO**^σ^**^–^C** | **K^+^12** (N^+^H_3_) | ion-polar |
| **Binding site 7**  Affinity (kcal/mol)  ‒3.9 | **APO_4_^–^** | **R^+^36**(=N^+^H_2_) | ionic |
|  | **BPO_4_^–^** | **N4**(NH_2_ ^σ+^), **R^+^58**(=N^+^H_2_) | ionic, ion-hydrogen |
|  | **1CO** ^σ^**^–^** | **R^+^36**(=N^+^H_2_) | ion-polar |
|  | **3C=O**^σ−^ | **T56**(OH ^σ+^) | hydrogen |
|  | **4C=O**^σ−^ | **R^+^58**(=N^+^H_2_) | ion-polar |
| **Binding site 8**  Affinity (kcal/mol)  ‒3.9 | **APO_4_^–^** | **K^+^35**(N^+^H_3_), **R36**(NH_pb_^σ+^) | ionic, ion-hydrogen |
|  | **BPO_4_^–^** | **K12**(NH_pb_^σ+^) | ion-hydrogen |
|  | **2CO**^σ^**^–^C** | **C38**(NH_pb_^σ+^) | hydrogen |
|  | **2C=O**^σ−^ | **K^+^18**(N^+^H_3_) | ion-polar |
|  | **5CO**^σ^**^–^C** | **K^+^12**(N^+^H_3_) | ion-polar |
|  | **5C=O**^σ−^ | **K^+^18**(N^+^H_3_) | ion-polar |
| **Binding site 9**  Affinity (kcal/mol)  ‒3.9 | **APO_4_^–^** | **R^+^36**(=N^+^H_2_) | ionic |
|  | **BPO_4_^–^** | **R^+^36**(=N^+^H_2_) | ionic |
|  | **4CO**^σ^**^–^C** | **N4**(NH_2_ ^σ+^) | hydrogen |
|  | **4C=O**^σ−^ | **R^+^58**(=N^+^H_2_) | ion-polar |

Hypothetical binding sites in CTI that bind to the phospholipid head group of CL as determined by AutoDock modeling. The table shows the complete list of amino acid residues in CTI that interact with the CL charged and polar groups at various binding sites. Pb in C=O_pb_^σ−^ or in NH_pb_^σ+^ denotes a peptide bond.
