# Supplementary material for: Naja naja oxiana Cobra Venom Cytotoxins CTI and CTII Disrupt Mitochondrial Membrane Integrity: Implications for Basic Three-Fingered Cytotoxins
Source: PLoS One. 2015 Jun 19;10(6):e0129248. doi: 10.1371/journal.pone.0129248 (PMC4474699; doi:10.1371/journal.pone.0129248)
Supplement: S7 Table — Hypothetical binding sites in CTII that bind to the phospholipid head group of PC as determined by AutoDock modeling. The table shows a complete list of amino acid residues in CTII that interact with the charged and polar groups of PC at various binding sites. Pb in C = Opb σ− or in NHpb σ+ denotes a peptide bond. (DOCX) [file pone.0129248.s009.docx]

| Binding site # | *PC polar groups* | *CTII amino acid residues* | *Bond type and orientation* |
| --- | --- | --- | --- |
| **Binding site 1**  Affinity (kcal/mol)  ‒2.8 | **PO_4_^–^** | **K^+^23**(N^+^H_3_) | ionic |
|  | **1CO**^σ^**^–^C** | **K^+^23**(N^+^H_3_) | ion-polar |
|  | **2CO**^σ^**^–^C** | **K^+^23**(N^+^H_3_) | ion-polar |
|  | **N^+^(CH_3_)_3_** | None | into solution |
| **Binding site 2**  Affinity (kcal/mol)  ‒2.6 | **PO_4_^–^** | **K^+^44**(N^+^H_3_), **S45**(OH ^σ+^), **S45**(NH_pb_^σ+^) | ionic, 2 ion-hydrogen |
|  | **N^+^(CH_3_)_3_** | None | into solution |
| **Binding site 3**  Affinity (kcal/mol)  ‒2.5 | **PO_4_^–^** | **S45**(OH ^σ+^), **S45**(NH_pb_ ^σ+^) | 2 ion-hydrogen |
|  | **1C=O** ^σ−^ | **K^+^44**(N^+^H_3_) | ion-polar |
|  | **2CO**^σ^**^–^C** | **S45**(NH_pb_ ^σ+^) | hydrogen |
|  | **2C=O** ^σ−^ | **K^+^50**(N^+^H_3_) | ion-polar |
|  | **N^+^(CH_3_)_3_** | **None** | into solution |
| **Binding site 4**  Affinity (kcal/mol)  ‒2.4 | **PO_4_^–^** | **S45**(NH_pb_ ^σ+^) | ion-hydrogen |
|  | **1CO**^σ^**^–^C** | **K^+^44**(N^+^H_3_) | ion-hydrogen |
|  | **2C=O** ^σ−^ | **S45**(OH ^σ+^) | hydrogen |
|  | **N^+^(CH_3_)_3_** | **None** | into solution |
| **Binding site 5**  Affinity (kcal/mol)  ‒2.3 | **PO_4_^–^** | **Y22**(OH ^σ+^) | ion-hydrogen |
|  | **1C=O** ^σ−^ | **K^+^5**(N^+^H_3_) | ion-polar |
|  | **2C=O** ^σ−^ | **K^+^18**(N^+^H_3_) | ion-polar |
|  | **2C=O** ^σ−^ | **K^+^18**(N^+^H_3_) | ion-polar |
|  | **N^+^(CH_3_)_3_** | **None** | into solution |
| **Binding site 6**  Affinity (kcal/mol)  ‒2.3 | **PO_4_^–^** | **K^+^18**(N^+^H_3_) | ionic |
|  | **1C=O** ^σ−^ | **Y22**(OH ^σ+^), **K^+^35**(N^+^H_3_) | hydrogen, ion-polar |
|  | **N^+^(CH_3_)_3_** | **None** | into solution |
| **Binding site 7**  Affinity (kcal/mol)  ‒2.3 | **PO_4_^–^** | **S45**(OH ^σ+^), **S45**(NH_pb_^σ+^) | 2 ion-hydrogen |
|  | **1CO**^σ^**^–^C** | **K^+^44**(N^+^H_3_) | ion-polar |
|  | **N^+^(CH_3_)_3_** | None | into solution |
| **Binding site 8**  Affinity (kcal/mol)  ‒2.3 | **PO_4_^–^** | **K^+^35**(N^+^H_3_) | ionic |
|  | **2CO**^σ^**^–^C** | **R^+^36**(=N^+^H_2_) | ion-polar |
|  | **2C=O** ^σ−^ | **R^+^36**(=N^+^H_2_) | ion-polar |
|  | **N^+^(CH_3_)_3_** | **P33**(C=O_pb_^σ−^) | ion-polar |
| **Binding site 9**  Affinity (kcal/mol)  ‒2.2 | **PO_4_^–^** | **N19**(NH_2_ ^σ+^) | ion-hydrogen, into solution |
|  | **2C=O** ^σ−^ | **G17**(NH_pb_ ^σ+^), **N19**(NH_2_ ^σ+^) | 2 hydrogen |
|  | **N^+^(CH_3_)_3_** | **N19**(C=O_pb_^σ−^) | ion-polar |

**S7 Table. Summary of amino acid residues in CTII that interact with PC.**

Hypothetical binding sites in CTII that bind to the phospholipid headg roup of PC as determined by AutoDock modeling. The table shows a complete list of amino acid residues in CTII that interact with the PC charged and polar groups at various binding sites. Pb in C=O_pb_^σ−^ or in NH_pb_^σ+^ denotes a peptide bond.
