# Supplementary material for: Naja naja oxiana Cobra Venom Cytotoxins CTI and CTII Disrupt Mitochondrial Membrane Integrity: Implications for Basic Three-Fingered Cytotoxins
Source: PLoS One. 2015 Jun 19;10(6):e0129248. doi: 10.1371/journal.pone.0129248 (PMC4474699; doi:10.1371/journal.pone.0129248)
Supplement: S8 Table — Hypothetical binding sites in CTII that bind to the phospholipid head group of PS as determined by AutoDock modeling. The table shows a complete list of amino acid residues in CTII that interact with the charged and polar groups of PS for various binding sites. Pb in C = Opb σ− or in NHpb σ+ denotes a peptide bond. (DOCX) [file pone.0129248.s010.docx]

| Binding site # | *PS polar groups* | *CTI amino acid residues & PS* | *Bond type and orientation* |
| --- | --- | --- | --- |
| **Binding site 1**  Affinity (kcal/mol)  ‒3.3 | **PO_4_^–^** | **K^+^23**(N^+^H_3_) | ionic |
|  | **COO^–^** | **NA** | into solution |
|  | **2C=O**^σ−^ | **K^+^23**(N^+^H_3_) | ion-polar |
|  | **N^+^H_3_** | **T56**(O^σ−^H) | ion-polar |
| **Binding site 2**  Affinity (kcal/mol)  ‒3.3 | **PO_4_^–^** | **K^+^2**(N^+^H_3_) | ionic, into solution |
|  | **COO^–^** | **T13**(OH ^σ+^), **T13**(NH_pb_^σ+^) | 2 hydrogen |
|  | **1CO**^σ^**^–^C** | **S11**(OH^σ+^) | hydrogen |
|  | **1** **C=O**^σ−^ | **S11**(OH^σ+^) | hydrogen |
|  | **N^+^H_3_** | **S11**(O^σ−^H), **S11**(C=O_pb_^σ−^) | 2 ion-polar |
| **Binding site 3**  Affinity (kcal/mol)  ‒3.2 | **PO_4_^–^** | **K^+^44**(N^+^H_3_), **S45**(NH_pb_^σ+^), **PS**(N^+^H_3_) | 3 ionic |
|  | **COO^–^** | **K^+^50**(N^+^H_3_) | ionic |
|  | **2C=O** ^σ−^ | **S45**(OH^σ+^) | hydrogen |
|  | **N^+^H_3_** | **PS**(PO_4_^–^) | ionic, into solution |
| **Binding site 4**  Affinity (kcal/mol)  ‒3.2 | **PO_4_^–^** | **S45**(OH ^σ+^), **K^+^50**(N^+^H_3_) | ionic, ion-polar |
|  | **COO^–^** | **C53**(NH_pb_ ^σ+^) | ion-hydrogen |
|  | **2C=O** ^σ−^ | **K^+^50**(N^+^H_3_) | ion-polar |
|  | **N^+^H_3_** | **K^+^44**(N^+^H_3_), **P43**(C=O_pb_^σ−^) | ionic repulsion, ion-polar, i.s. |
| **Binding site 5**  Affinity (kcal/mol)  ‒3.0 | **PO_4_^–^** | **S45**(OH ^σ+^), **S45**(NH_pb_ ^σ+^) | 2 ion-hydrogen |
|  | **COO^–^** | **C53**(NH_pb_ ^σ+^) | ion-hydrogen |
|  | **1CO**^σ^**^–^C** | **K^+^50**(N^+^H_3_) | ion-polar |
|  | **1C=O** ^σ−^ | **K^+^50**(N^+^H_3_) | ion-polar |
|  | **N^+^H_3_** | **K^+^44**(N^+^H_3_), **P43**(C=O_pb_^σ−^) | ionic repulsion, ion-polar, i.s. |
| **Binding site 6**  Affinity (kcal/mol)  ‒2.9 | **PO_4_^–^** | **S45**(NH_pb_ ^σ+^), **PS**(N^+^H_3_) | ion-hydrogen, ionic |
|  | **COO^–^** | **K^+^50**(N^+^H_3_) | ionic |
|  | **1CO**^σ^**^–^C** | **K^+^50**(N^+^H_3_) | ion-polar |
|  | **1C=O** ^σ−^ | **K^+^50**(N^+^H_3_) | ion-polar |
|  | **2CO**^σ^**^–^C** | **S45**(OH ^σ+^) | hydrogen |
|  | **N^+^H_3_** | **PS**(PO_4_^–^) | ionic, into solution |
| **Binding site 7**  Affinity (kcal/mol)  ‒2.8 | **PO_4_^–^** | **K^+^44**(N^+^H_3_), **S45**(NH_pb_^σ+^), **S45**(OH ^σ+^) | ionic, 2 ion-hydrogen |
|  | **COO^–^** | **C53**(NH_pb_ ^σ+^) | ion-hydrogen |
|  | **1CO**^σ^**^–^C** | **K^+^50**(N^+^H_3_) | ion-hydrogen |
|  | **N^+^H_3_** | NA | into solution |
| **Binding site 8**  Affinity (kcal/mol)  ‒2.8 | **PO_4_^–^** | **K^+^23**(N^+^H_3_) | ionic |
|  | **COO^–^** | NA | into solution |
|  | **1C=O** ^σ−^ | **K^+^23**(N^+^H_3_) | ion-polar |
|  | **NH_3_^+^** | **C59**(C=O_pb_^σ−^) | ion-polar |
| **Binding site 9**  Affinity (kcal/mol)  ‒2.8 | **PO_4_^–^** | **Y22**(OH ^σ+^) | ion-hydrogen |
|  | **COO^–^** | **K^+^35**(N^+^H_3_), **Y22**(OH^σ+^) | ionic, ion-hydrogen |
|  | **1CO**^σ^**^–^C** | **C38**(NH_pb_ ^σ+^) | hydrogen |
|  | **1C=O** ^σ−^ | **K^+^5**(N^+^H_3_) | ion-polar |
|  | **2C=O** ^σ−^ | **K^+^18**(N^+^H_3_) | ion-polar |
|  | **N^+^H_3_** | **Y22**(O^σ−^H) | ion-polar |

**S8 Table. Summary of amino acid residues in CTII that interact with PS**

Hypothetical binding sites in CTII that bind to the phospholipid head group of PS as determined by AutoDock modeling. The table shows a complete list of amino acid residues in CTII that interact with the PS charged and polar groups for various binding sites. Pb in C=O_pb_^σ−^ or in NH_pb_^σ+^ denotes a peptide bond.
