# Supplementary material for: Naja naja oxiana Cobra Venom Cytotoxins CTI and CTII Disrupt Mitochondrial Membrane Integrity: Implications for Basic Three-Fingered Cytotoxins
Source: PLoS One. 2015 Jun 19;10(6):e0129248. doi: 10.1371/journal.pone.0129248 (PMC4474699; doi:10.1371/journal.pone.0129248)
Supplement: S9 Table — Hypothetical binding sites in CTII that bind to the phospholipid head group of CL as determined by AutoDock modeling. The table shows a complete list of amino acid residues in CTII that interact with the charged and polar groups of CL at various binding sites. Pb in C = Opb σ− or in NHpb σ+ denotes a peptide bond. (DOCX) [file pone.0129248.s011.docx]

| Binding number | *CL polar groups* | *CTII amino acid residues* | *Bond type and orientation* |
| --- | --- | --- | --- |
| **Binding site 1** Affinity (kcal/mol)  ‒4.0 | **APO_4_^–^** | **K^+^5**(N^+^H_3_)**, C38**(NH_pb_^σ+^) | ionic, ion-hydrogen |
|  | **BPO_4_^–^** | **K^+^5**(N^+^H_3_) | 2 ionic, ion-hydrogen |
|  | **1CO**^σ^**^–^** | **K^+^5**(N^+^H_3_)**, K^+^12**(N^+^H_3_) | 2 ion-polar |
|  | **2CO**^σ^**^–^C** | **K^+^5**(N^+^H_3_) | ion-polar |
|  | **3CO**^σ^**^–^C** | **Y22**(OH ^σ+^) | hydrogen |
|  | **5C=O**^σ−^ | **L6**(NH_pb_ ^σ+^)**, V7**(NH_pb_ ^σ+^) | 2 hydrogen |
| **Binding site 2** Affinity (kcal/mol)  ‒4.0 | **APO_4_^–^** | **K^+^18**(N^+^H_3_) | ionic |
|  | **BPO_4_^–^** | **K^+^18**(N^+^H_3_) | ionic, into solution |
|  | **1CO**^σ^**^–^** | **K^+^18**(N^+^H_3_) | ion-polar |
|  | **3C=O**^σ−^ | **Y22**(OH ^σ+^) | hydrogen |
|  | **4CO**^σ^**^–^C** | **K^+^5**(N^+^H_3_) | ion-polar |
|  | **5CO**^σ^**^–^C** | **K^+^5**(N^+^H_3_)**, C38**(NH_pb_^σ+^) | ion-polar, hydrogen |
| **Binding site 3** Affinity (kcal/mol)  ‒3.8 | **APO_4_^–^** | **Y22**(OH ^σ+^) | ion-hydrogen, into solution |
|  | **BPO_4_^–^** | **K^+^18**(N^+^H_3_) | ionic |
|  | **2CO**^σ^**^–^C** | **K^+^35**(N^+^H_3_) | ion-polar |
|  | **4C=O**^σ−^ | **K^+^5**(N^+^H_3_) | ion-polar |
|  | **5CO**^σ^**^–^C** | **C38**(NH_pb_^σ+^) | hydrogen |
| **Binding site 4** Affinity (kcal/mol)  ‒3.7 | **APO_4_^–^** | **K^+^5**(N^+^H_3_) | ionic, into solution |
|  | **BPO_4_^–^** | **K^+^5**(N^+^H_3_)**, K^+^12**(N^+^H_3_) | 2 ionic |
|  | **2C=O**^σ−^ | **K^+^12**(N^+^H_3_) | ion-polar |
|  | **4CO**^σ^**^–^C** | **K^+^18**(N^+^H_3_) | ion-polar |
|  | **4C=O**^σ−^ | **K^+^18**(N^+^H_3_) | ion-polar |
|  | **5CO**^σ^**^–^C** | **C38**(NH_pb_^σ+^) | hydrogen |
|  | **5C=O**^σ−^ | **K^+^18**(N^+^H_3_) | ion-polar |
| **Binding site 5** Affinity (kcal/mol)  ‒3.7 | **APO_4_^–^** | **K^+^35**(N^+^H_3_), **Y22** | Ionic, ion-hydrogen |
|  | **1CO** ^σ^**^–^** | **Y51**( OH^σ+^) | hydrogen |
|  | **2C=O**^σ−^ | **K^+^35**(N^+^H_3_) | ion-polar |
| **Binding site 6** Affinity (kcal/mol)  ‒3.6 | **APO_4_^–^** | **K^+^5**(N^+^H_3_) | ionic |
|  | **BPO_4_^–^** | **K^+^35**(N^+^H_3_), **Y22**(OH^σ+^) | Ionic, ion-hydrogen |
|  | **2CO**^σ^**^–^C** | **K^+^18**(N^+^H_3_) | ion-polar |
|  | **3CO**^σ^**^–^C** | **K^+^18**(N^+^H_3_) | ion-polar |
|  | **4CO**^σ^**^–^C** | **K^+^35**(N^+^H_3_), **Y51**(OH^σ+^) | ion-polar, hydrogen |
|  | **4C=O**^σ−^ | **Y51**(OH ^σ+^) | hydrogen |
| **Binding site 6** Affinity (kcal/mol)  -3.5 | **APO_4_^–^** | **K^+^2**(N^+^H_3_)**, S11**(OH ^σ+^) | ionic, ion-hydrogen |
|  | **BPO_4_^–^** | **K^+^4**(N^+^H_3_), **K^+^58**(N^+^H_3_) | 2 ionic |
|  | **1CO**^σ^**^–^** | **K^+^58**(N^+^H_3_) | ion-hydrogen |
|  | **2C=O**^σ−^ | **S11**(OH ^σ+^) | hydrogen |
|  | **3C=O**^σ−^ | **T13**(OH^σ+^) | hydrogen |
|  | **5C=O**^σ−^ | **S11**(NH_pb_^σ+^) | hydrogen |
| **Binding site 8**  Affinity (kcal/mol)  ‒3.5 | **BPO_4_^–^** | **R^+^36**(=N^+^H_2_) | ionic, into solution |
|  | **1CO**^σ^**^–^** | **R^+^36**(=N^+^H_2_) | ion-polar |
|  | **2C=O^σ−^** | **K^+^23**(N^+^H_3_) | ion-polar |
|  | **3C=O^σ−^** | **R^+^36**(=N^+^H_2_) | ion-polar |
| **Binding site 9**  Affinity (kcal/mol)  ‒3.5 | **APO_4_^–^** | **Y22**(OH ^σ+^), **K^+^35**(N^+^H_3_) | ion-hydrogen, ionic |
|  | **3C=O^σ−^** | **C38**(NH_pb_^σ+^) | hydrogen |

**S9 Table. Summary of amino acid residues in CTII that interact with CL.**

Hypothetical binding sites in CTII that bind to the phospholipid head group of CL as determined by AutoDock modeling. The table shows a complete list of amino acid residues in CTII that interact with the CL charged and polar groups at various binding sites. Pb in C=O_pb_^σ−^ or in NH_pb_^σ+^ denotes a peptide bond.
